# The evidence for commercial house dust mite immunotherapy products: A pragmatic systematic review with narrative synthesis

**DOI:** 10.1016/j.jacig.2024.100255

**Published:** 2024-04-10

**Authors:** Timothy West, Constance H. Katelaris

**Affiliations:** a Campbelltown Hospital, Campbelltown, Australia; b Western Sydney University, Campbelltown, Australia

**Keywords:** House dust mite, allergic rhinitis, allergic asthma, allergen immunotherapy, sublingual immunotherapy, subcutaneous immunotherapy

## Abstract

House dust mite (HDM) allergen immunotherapy (AIT) has an established role in the treatment of perennial allergic rhinitis (AR) and allergic asthma (AA) triggered by HDM sensitization. We aimed to identify all double-blind, randomized, placebo-controlled trials of HDM AIT for the treatment of AR and AA in humans and to summarize the evidence for AIT products that are currently manufactured and available for clinical use.

A total of 56 eligible double-blind, randomized, placebo-controlled trials of HDM AIT for the treatment of AA and/or AR in humans fit the inclusion criteria and investigated a total of 14 commercial AIT products; together, the 56 studies enrolled a total of 14,619 patients. Of the 56 studies, 39 studies investigated the current manufacturer-recommended maintenance dose (MRMD) of the product, and 17 investigated other doses. We identified 39 studies (12,539 patients randomized) for 8 sublingual immunotherapy (SLIT) products and 17 studies (2,080 patients randomized) for subcutaneous immunotherapy products. For AR, 3 products, the ALK 12 standardized-quality (SQ-HDM) SLIT tablet, the ALK 6 SQ-HDM tablet, and the SG 300 index of reactivity SLIT tablet, had both dose-finding studies (DFSs) and phase III definitive studies (DSs) to demonstrate efficacy of the MRMD of the product. For AA, 2 products, the ALK 12 SQ-HDM SLIT tablet and the ALK 6 SQ-HDM tablet, had both DFSs and DSs for the MRMD. No subcutaneous immunotherapy product had a paired DFS and DS supporting the MRMD. A total of 30 studies of products no longer commercially manufactured were excluded. This study will help to inform clinical care and product selection for the treatment of HDM-induced AR and AA.

Allergic airway diseases are among the most common diseases globally, with allergic rhinitis (AR) and asthma estimated to affect 18% and 12% of adults, respectively.^1^^,^^2^ Approximately half of patients with asthma have allergic asthma (AA).^3^ *Dermatophagoides* species, principally *Dermatophagoides* *pteronyssinus* (DP) and *Dermatophagoides farinae* (DF), were identified as the major source of house dust mite (HDM) allergens in 1964^4^ and are the major global source of indoor allergens causing perennial AR and AA.^5^ The first double-blind, randomized, placebo-controlled trial (DBRPCT) of a commercially prepared HDM allergen immunotherapy (AIT), a subcutaneous immunotherapy (SCIT) product, was published by Smith and Pazzaro in 1972 (see Calderon et al)^6^; the first DBRPCT of HDM sublingual immunotherapy (SLIT) trial was published by Scadding et al in 1986 (see Larenas-Linnemann et al^7^); and the DBRPCT of a HDM allergoid AIT product was published by Passalacqua et al in 1998 (see Larenas-Linnemann et al^7^).

The AIT products used globally vary; the availability of products varies across countries, with a small number of products registered with each country’s medical regulation agency and a greater number available on a named patient product basis only.^8^ The European Medicines Agency (EMA) released guidelines on the regulation of AIT as a medical product in 2008^9^ and guidelines on the clinical development of AIT products in 2009.^10^ Both US and European regulators have advised that newly developed AIT products be evaluated as individual medical products requiring a phase II dose-finding study (DFS) and phase III definitive study (DS) for product registration.^10^^,^^11^ In 2008, the German Paul-Ehrlich-Institut initiated a process known as the Therapeutic Allergen Ordinance to retrospectively evaluate all AIT products for common allergens on the German market, and in doing so, it required a DFS and DS for each product.^12^ As of 2023, however, no HDM products had completed the therapeutic allergen ordinance process, and several manufacturers had withdrawn from it.^12^

The clinical performance of dust mite AIT is dependent on several production factors,^10^ including method of culturing dust mites, purification and processing of mite bodies and feces into extracts, and posttreatment modification of extracts.^13^ Consequently, total dust mite protein content and major and minor allergen content vary widely between products.^14^ The measurement of allergen content is not standardized and varies widely between AIT manufacturers; for this reason and because of their proprietary manufacturing and allergen modification steps, AIT products are not interchangeable, even when their reported major allergen contents are equal.^15^ Although recent systematic reviews have concluded that HDM SCIT and SLIT are both effective for AR and AA,^16^^,^^17^ these reviews do not identify which trials refer to currently available commercial AIT products; nor do they summarize the evidence for each AIT product individually. Furthermore, a 2013 review noted that several published DBRPCTs referred to products that were no longer commercially available, and some trials did not state the name of the HDM AIT product investigated or the dose and mite species administered.^6^ Thus, it is difficult for prescribers of AIT to know of the evidence base for each of the products available for use in the various countries.

To inform clinical care and product selection, our specific aims were to (1) identify all DBRPCTs of HDM AIT for the treatment of AR and AA in humans; (2) determine which studies refer to products that are currently manufactured and available for clinical use; (3) determine the product used in the various trials, the allergen mix, and the maintenance dose (in units) comparable to the current product information statement for each product; and (4) summarize the evidence on a per-product basis, noting specifically whether DFSs and phase III DSs have been performed for the current manufacturer-recommended maintenance dose (MRMD) of the product. For each product, we further aimed to describe whether there were published trials demonstrating short-term benefits of AIT, long-term benefits of AIT, and sustained symptom relief after cessation of AIT.

## Methods

The systematic review protocol was registered with the International Prospective Register of Systematic Reviews (PROSPERO) (registration no. CRD42023336291). In May 2022, searches were conducted in Embase, Medline, the European Clinical Trials Database (EudraCT) and the US clinical trials database CliniocalTrials.gov, with searches limited to the previous 22 years (from the year 2000 onward). For a copy of the search strategy, see the Supplementary Information in the Online Repository at www.jaci-global.org. For studies before the year 2000, the reference lists of 3 comprehensive systematic reviews were searched.^6^^,^^16^^,^^17^ The risk of bias from noninclusion of studies that have not been published was minimized by searching the European and US clinical trials registries for reports of studies conducted but not published in the searched databases.

The titles and abstracts of the retrieved studies were screened by 1 reviewer to identify studies that potentially met the inclusion criteria, and the same reviewer retrieved and assessed the full text of potentially eligible studies. Studies were included if they were DBRPCTs of HDM AIT for the treatment of AR or AS in humans, had at least 1 eligible treatment arm (see later), included clinical outcomes such as patient scoring systems (eg, combined symptom and/or medication scores) and/or objective measures such as challenge chamber tests, were of at least 12 weeks’ duration from initiation of AIT to clinical outcome assessment, had been published as full-length articles in academic journals, had results published to either the US or European clinical trials registries or had been prospectively registered in either the US or European clinical trials registry, and had results that could be obtained from the manufacturer when requested.

Treatment arms were considered eligible if the intervention in the arm was a commercially manufactured HDM AIT product and if the product and extract mix used in the arm was currently commercially available in at least 1 country worldwide. The treatment arms of products developed but not brought to market, noncommercial products, dust mite extracts mixed with other allergens (eg, mixtures of dust mite and grass pollen), and dust mite allergen mixes no longer commercially available were excluded.

After conducting a full-text review, we submitted queries regarding 61 studies to the manufacturers of the AIT products listed in each study to determine the AIT product name, allergen mix, dose used, and current availability of the product. The information obtained is summarized in Table E1 (available in the Online Repository at www.jaci-global.org). A standardized prepiloted form was used to extract data from the included studies for assessment of study quality and study synthesis. A single reviewer extracted the data. The extracted data items are listed in the Supplementary Materials. Risk of bias was not assessed.

Studies were categorized in 4 ways: (1) according to the disease treated (asthma, AR, or both); (2) depending on whether at least 1 arm included the current MRMD of the AIT product under investigation in that article; (3) as DFSs (phase II), DSs (phase III), or exploratory studies (ESs); and (4) as showing (according to the EMA Guidelines^10^) either short-term benefit (STB), long-term benefit (LTB), or disease-modifying treatment (DMT). We defined DFSs as those studies that included 2 or more intervention arms of the same product at different doses and had prespecified end points for clinical efficacy. We defined DSs as those studies having prespecified outcomes for treatment efficacy on clinical grounds according to the EMA Guidelines,^10^ a prespecified power calculation, and at least 100 patients randomized to the active treatment arm. All other studies were considered ESs. According to our definitions, a study could be both a DFS and a DS. Studies were categorized as examining STB if they were designed to demonstrate an improvement in clinical outcomes during AIT treatment and had a duration of less than 24 months (including open-label follow-up). Studies were categorized as examining LTB if they were designed to demonstrate clinical outcomes during AIT treatment and had a duration of 24 months or longer. Studies were categorized as examining DMT if they assessed clinical outcomes following at least 12 months after cessation of AIT. According to our definitions, all studies were categorized as examining either STB or LTB but not both and could be additionally categorized as examining DMT. Long-term follow-up publications were identified during the search process and were incorporated into these analyses.

After categorizing all of the studies in these 4 ways, we identified products for which DFSs and/or DSs at the MRMD had been identified. In the case of products for which a paired DFS and DS set had been performed to establish efficacy of the products’ MRMDs, we performed descriptive narrative synthesis to summarize the totality of evidence for those products. Because of study heterogeneity and the small number of studies identified for most AIT products, meta-analysis was not possible. We further noted studies categorized as either DFSs or DSs that included long-term outcomes (ie, as examining either LTB or DMT).

During data extraction, it became evident that multiple products do not have a generic name. Because our study aimed to summarize the data for each product, it was important that we be able to identify the product investigated in each study. We therefore created a short name for each AIT product based on its current manufacturer’s name; see column 1 of Table I. This was considered preferable to using either the brand name or units of measurement for each product, because both can change over time and vary between countries.

**Table I:** Currently commercially available AIT products for which randomized, double-blind, placebo-controlled trials were identified for the treatment for AR and/or asthma in humans using HDM extracts

Product name in this review	Generic name; brand and/or alternate names; manufacturer	Unit of measurement and major allergen content	Allergen formulation and commercially available presentation	Duration of initiation protocol	Maintenance dose	Mite species/composition for which studies identified
SLIT products						
ALK 12 SQ-HDM SLIT tablet	12 SQ-HDM tablet; Acarizax (Europe and Australia), Odactra (US), Mitizax (former), MK-8237; ALK-Abelló A/S, Denmark	SQ-HDM (formerly DU); 1 SQ-HDM = 1 DU^56^^,^^57^ The 12-SQ HDM dose contains 30 μg of major allergen, comprising approximately 15 μg of group 1 allergens (Der f 1 and Der p 1 combined) and approximately 15 μg of group 2 allergens (Der f 2 and Der p 2 combined)^57^	Native allergen Lyophilized tablet, 1 formulation: 12 SQ-HDM	1 d	1 × 12 SQ-HDM tablet SL daily	DP 50%–DF 50% mix
ALK 6 SQ-HDM SLIT tablet (marketed in Japan only)	6 SQ-HDM tablet; Miticure (Japan), MK-8237; ALK-Abelló A/S, Denmark	SQ-HDM (formerly DU [1 SQ-HDM = 1 DU^28^^,^^57^])	Native allergen Lyophilized tablet, 1 formulation: 6 SQ-HDM	1 d	1 × 6-SQ-HDM tablet SL daily	DP 50%–DF 50% mix
SG 300 IR SLIT tablet	300 IR tablet; Orylmyte (Germany); Actair (outside Germany), STG320; S-524101; Stallergenes Greer International AG; Switzerland	IR One 300-IR tablet contains 8-19 μg of Der p 1 and 39-79 μg of Der f 1^58^	Native allergen Lyophilized tablet, 2 formulations: 100 IR and 300 IR	3 d	1 × 300 IR tablet SL daily	DP 50%–DF 50% mix
SG SLIT liquid	300 IR/mL SLIT spray; Staloral; Stallergenes Greer International AG; Switzerland	IR 1 mL of 100-IR allergen extract of the DP 50%–DF 50% mix formulation contains 26.3 μg of Der p 1 and 63.4 mg of Der f 1^59^	Native allergen Liquid in pump spray bottle; 200 μg/pressure pump spray bottle. Available in 2 formulations: 10 IR/mL and 300 IR/mL. Previously available with 100 μg/pressure pump spray bottle and 50 μg/drop dropper	10 d	300 IR daily (5 × 200 μg of pressures of 300 IR/mL formulation) daily	DP 50%–DF 50% mix DP 100%∗
SG FDA-standardized extracts	Standardized mite *D farinae; D farinae* concentrate; Stallergenes Greer International AG; Switzerland†	AU 1 mL of the 10,000-AU/mL extract contains 169 mg/mL of Der f 1^18^	Native allergen in 50% glycerinated saline diluent Liquid in vial; 2 formulations: 5,000 AU/mL and 10,000 AU/mL (10-, 30-, or 50-mL vials). The liquid is diluted and packaged into pump spray bottles by clinicians or their associates	PI does not specify duration of initiation protocol^60^; practice varies.^8^ The initiation protocol of the high-dose arm of the study included in this review was 28 d^18^	PI does not specify MRMD. Dosages vary by mode of administration, and by individual response and tolerance Doses used in the study identified in this review were: high-dose: 4200 AU/d; low-dose: 60 AU/d^18^	DF 100%∗
WBCL DF SLIT liquid	*D farinae* drops; Chanllergen; Zhejiang Wolwo Bio-Pharmaceutical Co Ltd; China	DF protein concentration (μg/mL)	Native allergen Liquid in dropper bottle; 1 drop = 40 μL; 5 formulations (DF protein concentration): 1 μg/mL, 10 μg/mL, 100 μg/mL, 333 μg/mL, and 1,000 μg/mL)	3 wk using vials 1 to 3	Patients < 14 y: 1000 μg (3 drops of 333 μg/mL formulation) daily Patients ≥ 14 y: 2000 μg (2 drops of 1000 μg/mL formulation) daily	DF 100%
Lofarma SLIT tablet	Lais Mites sublingual tablets; Lofarma SpA; Italy	AU or UA One 1000-AU tablet contains 2.7 μg of group 1 mite allergens^61^	Monomeric allergoid Tablet, 2 formulations: 300 AU and 1,000 AU	4 d	1 × 1000 AU tablet SL ≥2 times per wk	DP 50%–DF 50% mix
Inmunotek SLIT liquid	SLIMtdc; Inmunotek SL; Spain	mTU (mannan-conjugated TU) 3000 mTU contains 5 μg of group 1 mite allergens (Der p 1 and Der f 1)^43^	Polymerized allergen bound to mannan Liquid in spray bottle; 1 formulation: 3,000 mTU/mL	1 d	600 mTU (2 × 100 μL sprays) SL daily	DP-DF mix
SCIT products						
Allergopharma allergoid SCIT	Acaroid; Allergopharma GmbH & Co KG; Germany	TU; 1 PNU = 3.333 TU^41^ The 10,000-TU/mL DP solution contains 12 μq/mL of Der p 1 and 10μg/mL Der p 2^62^	Monomeric allergoid liquid 2 formulations: 1,000 TU/mL (strength A) and 10,000 TU/mL (strength B)	8 injections 7-14 d apart	6,000 TU (0.6 mL of 10,000 TU/mL solution) every 4-8 wk†	DP 100%∗
ALK alum SCIT	ALK-depot SQ 503 *Dermatophagoides pteronyssinus*; Alutard SQ; ALK-Abelló A/S; Denmark	SQ-U The 100,000 SQ-U/mL formulation contains 9.8 μg/mL of Der p 1^42^	Native allergen, alum-precipitated liquid 4 formulations: 100, 1,000, 10,000, and 100,000 SQ-U/mL	16 injections, 7-14 d apart *or* 7 injections, 7 ± 2 d apart *or* 11 injections over 7 d, spread 7 ± 2 d apart (cluster)	100,000 SQ-U (1 mL of 100,000 SQ-U/mL formulation) every 4-8 wk	DP 100%∗
Roxall alum SCIT	Allergovac; Roxall Medizin GmbH; Germany (acquired from Bial Industrial Farmacéutica SA; Spain)	TSU (treatment standardized unit); some publications use SPT units; the manufacturer advised that 0.125 SPT is equal to 1 TSU (see Table E2)	Native allergen, alum-precipitated liquid 3 formulations: 10, 100, and 1,000 TSU/mL	9 weekly injections (conventional) *or* 6 weekly injections (fast)	1000 TSU (1 mL of 1000 TSU/mL formulation) every 4 wk	DP 100%∗
LETI allergoid SCIT	Depigoid; LETI Pharma, SLU; Spain	DPP^63^; the manufacturer advised that the 100-DPP/mL vial of the 100% DP formulation contains 14.25 μg of Der p1/mL and 8.61 μg Der p2/mL; see Table E2	Glutaraldehyde allergoid, polymerized, alum-precipitated liquid 1 formulation: 100 DPP/mL	6 weekly injections (conventional) *or* 2 injections on 1 d (rush)	50 DPP (0.5 mL of 100 DPP/ML formulation) every 30 d	DP-DF mix DP 100%∗
Allergopharma alum SCIT	Novo-Helisen Depot; Allergopharma GmbH & Co KG; Germany	TU	Native allergen, alum-precipitated liquid 3 formulations: 50 TU/mL, 500 TU/mL and 5,000 TU/mL	14 injections every 7-14 d	5000 TU (1 mL of 5000 TU/mL formulation) every 4-6 wk	DF 50%–DP 50% mix
HAL Allergy allergoid SCIT	Purethal Mites; HAL Allergy BV; The Netherlands	AUeq; the 20,000-AUeq/mL formulation contains 14 μg/mL of group 1 allergens (Der p 1 and Der f 1 combined) and 20 μg/mL of group 2 allergens (Der p 2 and Der f 2 combined)^40^	Allergoid, alum-precipitated liquid 1 formulation: 20,000 AUeq/mL	6 weekly, then 3 fortnightly injections (conventional) *or* 3 weekly, then 3 fortnightly injections (rush)	10,000 AUeq (0.5 mL of 20,000 AUeq/mL formulation) every 4 wk†	DF 50%–DP 50% mix

*AUeq*, Allergenic unit-equivalent; *Der f, Dermatophagoides farinae*; *Der p, Dermatophagoides pteronyssinus*; *DF, Dermatophagoides farinae*; *DP*, *Dermatophagoides pteronyssinus*; *DPP*, Depigmented polymerized unit; *DU*, Development unit; *mTU*, Mannan-conjugated therapeutic unit; *PI,* product information; *PNU*, Protein-nitrogen unit; *SL*, sublingually; *SPT*, Skin prick test; *SQ-U,* standardized quality unit; *TSU*, treatment-standardized unit; *TU*, therapeutic unit; *UA*, unità allergica.

Studies that enrolled patients with both AR and AA and reported clinical outcomes for both conditions are listed twice.

∗ Formulations of other mite species or mixes of mite species are marketed, but no randomized controlled trials were found for these formulations.

† Although companies other than Stallergenes-Greer also manufacture bulk FDA-standardized extracts, the only studies we identified used extract manufactured by Stallergenes-Greer.

## Results

**Fig 1:**
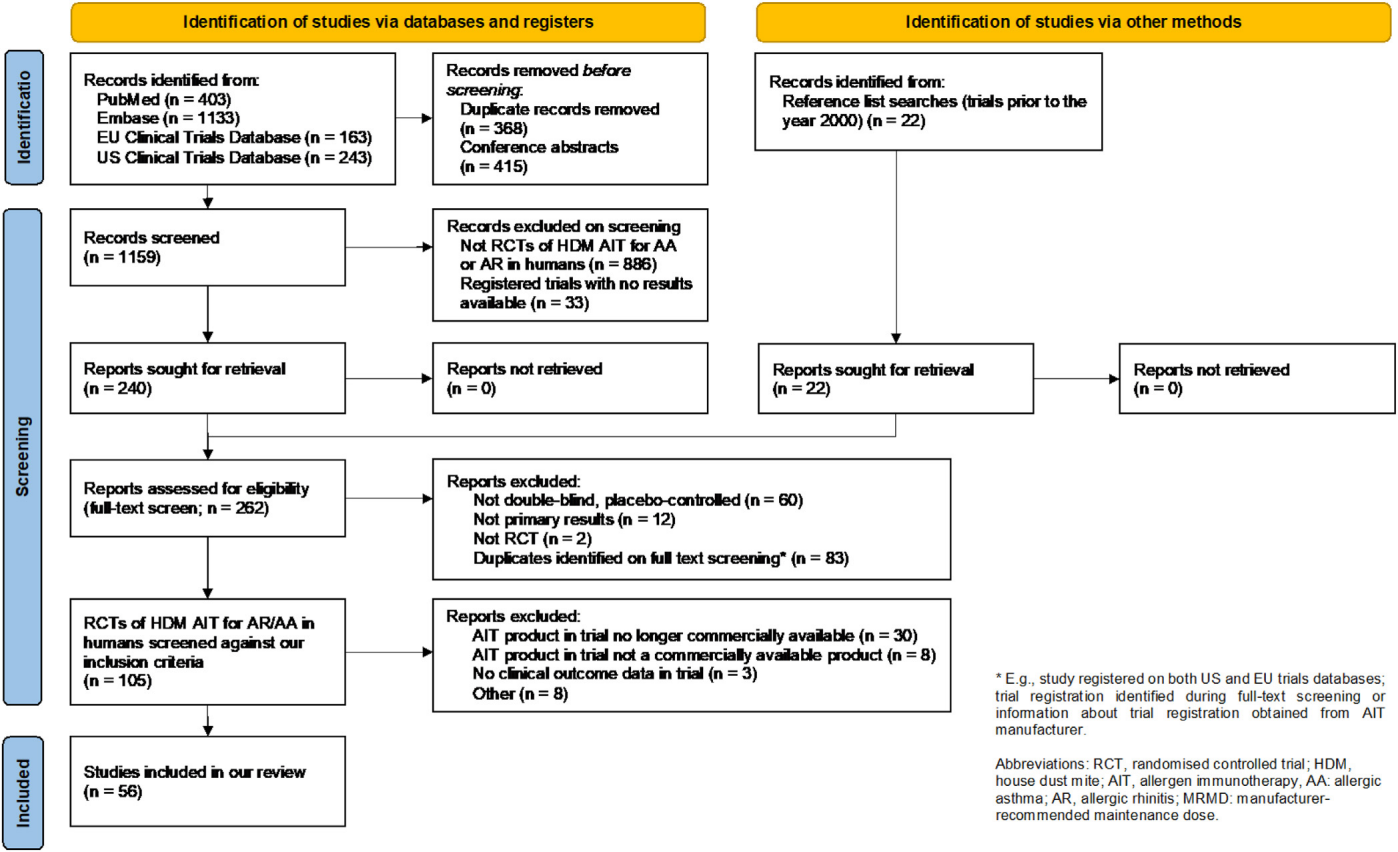
Study selection (Preferred Reporting Items for Systematic Reviews and Meta-Analyses [PRISMA] diagram). *RCT,* Randomized controlled trial.

We identified 105 DBRPCTs of HDM AIT for the treatment of AA and/or AR in humans. From among these, we excluded 49 studies: 30 studies of products that are no longer commercially manufactured, 8 studies of noncommercialized extracts or products that were never brought to market, 3 studies that did not report clinical outcomes (eg, laboratory results only), and 8 that were excluded for other reasons. The search strategy is shown in Fig 1. Notable studies excluded, including the reason for their exclusion, are in listed Table E2 (available in the Online Repository at www.jaci-global.org).

The remaining 56 studies investigated a total of 14 commercial AIT products that are currently available in at least 1 market worldwide; together, these 56 studies randomized a total of 14,619 patients. For SLIT, there were 39 studies that examined 8 products and randomized a total of 12,539 patients. For SCIT, there were 17 studies that examined 6 products and randomized a total of 2080 patients. The products identified and the characteristics thereof are summarized in Table I. Characteristics were obtained from the published studies and the manufacturer's product information for each product. The studies identified for each product, their categorization according to our criteria (DFS, DS, or ES), and the total number of patients investigated for each AIT product are shown in Table II.

**Table II:** Summary of studies identified for currently commercially available HDM immunotherapy products for the treatment of AR and/or AA in humans

Product name in this review; brand names)	Studies in AR			Studies in AA			Total in AA and AR	
	First name and year of publication (DS, DFS)∗	Total No. of patients randomized	No. of patients randomized to MRMD	First name and year of publication (DS; DFS)∗	Total No. of patients randomized	No. of patients randomized to MRMD	No. of patients randomized	No. of patients randomized to MRMD
SLIT products								
ALK 12 SQ-HDM SLIT tablet (Acarizax; Odactra) ALK 6 SQ-HDM SLIT tablet (Miticure)	Studies of both 6 SQ-HDM and 12 SQ-HDM dose Demoly et al, 2016 (DFS, DS)^19^ Nolte et al, 2015 (DFS)^21^ Okubo et al, 2017 (DFS, DS)^23^ Studies of 12 SQ-HDM but not 6 SQ-HDM dose Nolte et al, 2016 (DS)^22^ Bozek et al, 2022^26^^,^† Studies of 6 SQ-HDM but not 12 SQ-HDM dose Mosbech et al, 2014 (DFS, DS)^27^,† Masuyama et al, 2018 (DS)^20^	4638	12 SQ-HDM dose: 1432 6 SQ-HDM dose: 1073	Studies of both 6 SQ-HDM and 12 SQ-HDM dose Tanaka et al, 2020 (DFS, DS)^25^ Virchow et al, 2016 (DFS, DS)^24^ Studies of 12 SQ-HDM but not 6 SQ-HDM dose Bozek et al, 2022^26^^,^† Studies of 6 SQ-HDM but not 12 SQ-HDM dose Mosbech et al, 2014 (DFS, DS)^27^^,^†	2296	12 SQ-HDM dose: 576 6 SQ-HDM dose: 705	6298	12 SQ-HDM dose: 1991 6 SQ-HDM dose: 1622
SG 300 IR SLIT tablet (Actair; Orylmyte)	Studies of MRMD Bergmann et al, 2014 (DFS, DS)^29^ Demoly et al, 2021 (DS)^33^ Okamoto et al, 2017 (DFS, DS)^30^ Okamoto et al, 2019 (DS)^31^ Roux et al, 2016 (DFS)^32^ Studies of other doses: Tonnel et al, 2004^35^	3909	1599	Studies of MRMD Pham-Thi et al, 2007^34^ Studies of other doses SG 2013-000487-28 (DFS, DS)^36^	497	55	4406	1654
SG SLIT liquid (Staloral)	Studies of MRMD: none identified Studies of other doses Aydogan et al, 2013^59^ Bahceciler et al, 2001^64^^,^† Bozek et al, 2013^65^ Guez et al, 2000^66^ Mortemousque et al, 2003^67^ O'Hehir et al, 2009^68^ Potter et al, 2015^69^ Tseng et al, 2008^70^	426	0	Studies of MRMD Niu et al, 2006^71^ Wang et al, 2014 (DS)^37^ Studies of other doses Bahceciler et al, 2001^64^^,^† Bousquet et al, 1999^72^ Lue et al, et al, 2006^73^	714	378	1125	378
SG FDA-standardized extracts	Studies identified: Bush et al, 2011^18^ (The PI for this product does not specify a MRMD.)	31	0	No studies identified	—	—	—	—
WBCL DF SLIT liquid (Chanllergen)	Studies of MRMD: Chen et al, 2017^74^ Luo et al, 2014^75^ Wang et al, 2016^76^ Wen 2018	204	102	Studies of MRMD Tian et al, 2014^77^	60	30	264	132
Lofarma SLIT tablet (Lais Mites)	Studies of MRMD: Passalacqua et al, 2006^53^ Studies of other doses Huser et al, 2017 (DFS)^52^ Passalacqua et al, 1998^48^	219	34	No studies identified	—	—	219	34
Inmunotek SLIT liquid (slimTDC)	Studies of MRMD Nieto et al, 2022 (DFS)^43^	196	19	No studies identified	—	—	196	19
Total patients undergoing SLIT		9623	4259	Total patients undergoing SLIT	3567	1744	12,539	*5830*
SCIT products								
Allergopharma allergoid SCIT (Acaroid)	Studies of MRMD Allergopharma 2006-000934-11^78^ Dokic et al, 2005^38^ Studies of other doses Allergopharma 2015-000188-15 (DS)^50^^,^†	572	71	Studies of MRMD Allergopharma 2004-003892-35^46^ (adult patients only) Jutel et al, 2018 (DFS)^41^ Studies of other doses Allergopharma 2015-000188-15 (DS)^50^^,^†	634	62	782	133
ALK alum SCIT (ALK-depot SQ)	Studies of MRMD Varney et al, 2003^79^ Xian et al, 2020^80^	106	47	Studies of MRMD Blumberga et al, 2006^42^ Wang et al, 2006^81^		92	292	139
Roxall alum SCIT (Allergovac)	Studies of MRMD Moreno et al, 2016 (DFS)^39^	136	17	No studies identified	—	—	136	17
LETI allergoid SCIT (Depigoid)	Studies of MRMD Garcia-Robaina et al, 2006 (DP-DF mix)^82^^,^†	64	32	Studies of MRMD Garcia-Robaina et al, 2006 (DP-DF mix)^82^^,^† Ameal et al,^49^ 2005 (DP 100%)	127	64	127	64
Allergopharma alum SCIT (Novo-Helisen Depot)	Studies of MRMD Allergopharma - NCT00263549^83^ Yukselen et al, 2012^84^^,^†	167	80	Studies of MRMD Yukselen et al, 2012^84^^,^†	32	10	167	80
HAL Allergy allergoid SCIT (Purethal Mites)	Studies of MRMD Bozek et al, 2017^40^ Pfaar et al, 2016 (DFS)^45^ Studies of other doses HAL Allergy 2016-000051-27 (DS)^51^	576	89	No studies identified	—	—	576	89
Total patients undergoing SCIT		1621	336	Total patients undergoing SCIT	979	227	2080	521

*DF, Dermatophagoides farinae*; *DP*, *Dermatophagoides pteronyssinus*; *PI*, product information.

∗ According to our criteria, studies not annotated as DSs or DFSs were ESs.

† Study recruited patients with both AA and AR.

Of the 56 studies, 39 included an arm with an AIT dose matching the MRMD of a currently commercially available AIT product, 16 did not include an arm with the current MRMD of any commercially available products, and 1 study examined the US Food and Drug Administration (FDA)-standardized extracts, for which no MRMD is specified.^18^ Studies of doses other than the current MRMDs are listed in Table E3 (in the Online Repository at www.jaci-global.org). The remaining studies, comprising studies of the current MRMDs of commercially AIT products and the 1 study of the FDA-standardized extracts, are summarized in Table III (SLIT for the treatment of AR), Table IV (SLIT for the treatment of AA), Table V (SCIT for the treatment of AR), and Table VI (SCIT for the treatment of AA).

**Table III:** DBRPCTs of MRMDs of current commercially available SLIT products for HDM-induced AR

Lead author; year of publication; trial registration; classification according to our criteria	Study design; sites	Inclusion criteria: participant age; AR characterization	Population age (y; mean ± SD or median [range])	Sensitization	No arms; total patients randomized; MRMD: ITT/PP; Pl: ITT/PP	Maintenance dose and schedule; duration of blinded treatment; subsequent unblinded or untreated follow-up	Prespecified primary outcome	Other clinical outcomes of note	Study comments and limitations
12 SQ-HDM tablet; Acarizax (Europe and Australia), Odactra (US), ALK-Abelló A/S, Denmark; standardized mix of DP (50%) and DF (50%); marketed maintenance dose: 1 × 12 SQ-HDM tablet SL daily									
Bozek et al, 2022 (AR results)^26^ ES; STB	2 sites; Poland	Age ≥ 18 y; diagnosed local AR and concomitant GINA mild-to-moderate chronic asthma; clinical symptoms indicating HDM allergy; NPT result positive with DP and DF allergens	In MRMD arm: 33.7 ± 3.9; in Pl arm: 35.9 ± 4.2	Positive NPT result to DP and DF, with negative SPT result and sIgE to DP, DF other common aeroallergens	2 arms, 32 patients; in MRMD arm: 17/16; in Pl arm: 15/14	1 × 12 SQ-HDM tablet SL daily; 12 mo of maintenance; efficacy assessments at 6 and 12 mo	Primary outcome not prespecified	Mean monthly TRSS at 12 mo: 0.65 ± 0.33 with MRMD vs 1.75 ± 1.03 with Pl (*P* < .05, S) Mean monthly TMS (AR and AA medications combined) at 12 mo: 1.07 ± 0.56 with MRMD vs 2.89 ± 2.56 with Pl (*P* < .05, NS)	Study included local AR and AA. Study was not prospectively registered. Primary outcome was not prespecified. AR outcomes did not include CSMS. AA outcome was symptom score only. Outcomes were compared an a per-arm basis with respect to baseline period, but comparison of MRMD and Pl arms was not performed
Demoly et al, 2016^19^ (US: NCT01454544; Europe: 2011-002277-38) DFS; DS; STB	100 sites in 12 countries; Europe	Age 18- 65 y; moderate-to-severe HDM-induced AR symptoms (TRSS ≥ 6 or 5 with 1 severe symptom); use of AR medication during ≥8 d of the 15-d baseline period	Overall: 32.3 ± 10.9	Overall: 32% monosensitized to HDM Overall cosensitizations: grass pollen, 41%; cat, 41%; dog, 28%; birch pollen, 22%	3 arms, 992 patients; in MRMD arm: 318/284; in Pl arm: 338/296	1 × 12 SQ-HDM tablet SL daily; 10 mo of treatment maintenance, then 2 mo of efficacy assessment	Positive outcome: TCRS averaged over the last 8 wk of treatment: 5.53 with MRMD vs 6.76 with PI (*P* = .001, S); relative difference from Pl: 18%	Statistical improvement in the following secondary outcomes with MRMD vs with Pl: rhinitis symptoms and medication scores, RQLQ, symptom-free d, and combined rhinoconjunctivitis score. No statistical improvement in combined conjunctivitis score or global evaluation (% improved)	—
Nolte et al, 2015^21^ (US: NCT01644617; Europe: 2012-001855-38) DFS; STB; LTB	Single site; Vienna, Austria	Age ≥ 18 y; HDM-induced AR with or without conjunctivitis of ≥ 1 y with or without asthma; positive VCC result using DP-DF mixture	In MRMD arm: 28 (18-58); in Pl arm: 27 (19-43)	Monosensitized to HDM: in MRMD arm: 5/42 (12%); in Pl arm: 7/41 (17%) In MRMD arm, cosensitizations were birch, 52%; timothy grass, 67%; cat, 45%; dog, 48%	3 arms, 124 patients; in MRMD arm: 42/36; in Pl arm 41/34	1 × 12 SQ-HDM tablet SL daily; 24 wk of treatment maintenance, then 2 wk of follow-up	Positive outcome: TNSS at 24 wk: 3.83 with MRMD vs 7.45 with Pl (*P* < .05, S); relative difference from Pl: 49%	Improvement in RQLQ at 24 wk: 26% reduction with MRMD vs with Pl (*P* < .05) LTB 12 mo following cessation of AIT demonstrated in Zieglmayer et al 2016^85^	Primary outcome, and all symptom scores, evaluated in challenge chamber. Symptom scores outside challenge chamber (ie, day-to-day clinical scores) not assessed
Nolte et al, 2016^22^ (US: NCT01700192) DS; STB	182 sites; US and Canada	Age ≥ 12 y; HDM-induced AR with or without conjunctivitis of ≥ 1 y; DSS ≥ 6, or > 5 with 1 severe symptom	In MRMD arm: 35 ± 14; in Pl arm: 35 ± 14	Monosensitized to HDM; in MRMD arm: 184/741 (25%); in Pl arm: 171/741 (23%)	2 arms, 1482 patients; in MRMD arm: 741/561; in Pl arm 741/613	1 × 12 SQ-HDM tablet SL daily; 52 wk of maintenance	Positive outcome: average TCRS during the last 8 wk of treatment : 4.1 with MRMD vs 4.95 with Pl *(P* < .001, S); relative difference from Pl: 17%	Statistical improvement in the following secondary outcomes with MRMD vs with Pl: average rhinitis daily symptom score, conjunctivitis symptom score, RQLQ. No difference with MRMD vs with Pl in rhinitis daily medication score	—
Okubo et al, 2017^23^ (Japan: JapicCTI-121848) DFS; DS; STB	90 sites; Japan	Age 12-64 y; moderate-to-severe HDM-induced AR with ≥1-y medication history of AR. AR symptom score ≥ 7 on at least 7 of the 14-d run-in period without any symptomatic treatment	Overall: 26.9 ± 11.9	Overall: 20.9% monosensitized to HDM Overall cosensitizations were Japanese cedar pollen, 67%; Japanese cypress pollen, 34%; cat, 26%; orchard grass, 23%; dog, 15%	3 arms, 946 patients; in MRMD arm: 314/281; in Pl arm: 319/285	1 × 12 SQ-HDM tablet SL daily; 52 wk of treatment maintenance	Positive outcome: TCRS during the last 8 wk: 4.14 with MRMD vs 5.15 with Pl (*P* = .001, S); relative difference from Pl: 19%	Statistical improvement in the following secondary outcomes with MRMD vs with Pl: AR symptom score, conjunctivitis symptom score. No improvement in AR medication score with MRMD vs with Pl. Subanalysis of adolescent population (12-17 y) consistent with overall study results	—
6 SQ-HDM tablet; Miticure (Japan); ALK-Abelló A/S, Denmark; standardized mix of DP (50%) and DF (50%); marketed maintenance dose: 1 × 6 SQ-HDM tablet SL daily									
Demoly et al, 2016^19^ (US: NCT01454544; Europe: 2011-002277-38) DFS; DS; STB	100 sites; 12 countries; Europe	Age 18-65 y; moderate-to-severe HDM-induced AR symptoms (TRSS ≥6 or 5 with 1 severe symptom); use of AR medication during ≥8 d of the 15-d baseline period	Overall: 32.3 ± 10.9	Overall: 32% monosensitized to HDM Overall cosensitizations were grass pollen, 41%; cat, 41%; dog, 28%; birch pollen, 22%	3 arms, 992 patients; in MRMD arm: 336/297; in Pl arm: 338/296	1 × 6 SQ-HDM tablet SL daily; 10 mo of treatment maintenance, then 2 mo of efficacy assessment	Positive outcome: TCRS averaged over the last 8 wk of treatment: 5.58 with MRMD vs 6.76 with PI (*P* = .002, S); relative difference from Pl: 18%	Statistical improvement in the following secondary outcomes with MRMD vs with Pl: rhinitis symptoms and medication scores, symptom-free days, and combined rhinoconjunctivitis score. No statistical improvement in RQLQ, combined conjunctivitis score, combined rhinoconjunctivitis score or global evaluation (% improved)	—
Masuyama et al, 2018^20^ (Japan: JapicCTI-152953) DS; STB	66 sites; Japan	Age 5-17 y; moderate-to-severe HDM AR; medication history for HDM AR lasting ≥1 y; AR symptom score of ≥7 for ≥7 d of the 14-d run-in period without any symptomatic treatment	In MRMD arm: 10.8 ± 2.9; in Pl arm: 10.7 ± 3.1	Monosensitized to HDM: in MRMD arm: 51/227 (23%); in Pl arm: 74/231 (32%)	2 arms, 458 patients; in MRMD arm: 227/209; in Pl arm: 231/218	1 × 6 SQ-HDM tablet SL daily; 52 wk of treatment maintenance	Positive outcome: TCRS during the last 8 wk: 4.07 with MRMD vs 5.37 with Pl (*P* < .001, S); relative difference from Pl: 24%	Statistical improvement in the following secondary outcomes with MRMD vs with Pl: AR symptom score, AR medication score, combined conjunctivitis score, combined symptom score, conjunctivitis medication score, symptom-free days, JRQLQ	—
Mosbech et al, 2015^28^ (AR substudy of Mosbech H et al, 2014^27^) (US: NCT00389363; Europe: 2006-001795-20) DFS; DS; STB	81 sites; Europe	Age > 14 y; TCRS > 0 during 4-wk baseline period; mild-to-moderate HDM AA requiring ICS use (100-800 mg/d of budesonide or equivalent)	Graphical data only; see article	Overall 28% monosensitized to HDM	4 arms; substudy of 489/604 patients in trial of AA; in MRMD arm: 134/117; in Pl arm: 107/95	1 × 6 SQ-HDM tablet SL daily; 1 y of maintenance treatment	Primary outcome of this study relates to AA, not AR, and is listed in Table IV	TCRS averaged over last 4 wk of trial 1.93 with MRMD vs 2.71 with Pl (*P* = .036, S); relative difference 29% RQLQ absolute difference –.024 (95% CI = –0.43 to –0.05) with MRMD vs with Pl (*P* = .036)	Primary outcome of the study was regarding AA 18% of overall population had no AR symptoms at baseline. Article describes outcomes of *post hoc* analysis of patients with TCRS > 0 during screening period
Nolte et al, 2015^21^ (US: NCT01644617; Europe: 2012-001855-38) DFS; STB; LTB	Single site; Vienna, Austria	Age ≥ 18 y; HDM-induced AR with or without conjunctivitis of ≥ 1 y with or without asthma; positive VCC result using DP-DF mixture	In MRMD arm: 27 (20-48); in Pl arm: 27 (19-43)	Monosensitized to HDM: in MRMD arm: 4/41 (10%); in Pl arm: 7/41 (17%) Cosensitizations in MRMD arm: birch, 59%; timothy grass, 78%; cat, 56%; dog, 54%	3 arms, 124 patients; in MRMD arm: 41/36; in Pl arm: 41/34	1x 6 SQ-HDM tablet SL daily; 24 wk of treatment maintenance, then 2 wk of follow-up	Positive outcome: TNSS in VCC at 24 wk: 5.47 with MRMD vs 7.45 with Pl (*P* < .05, S); relative difference from Pl: 49%	No improvement in RQLQ at wk 24 with MRMD vs with Pl LTB 12 mo following cessation of AIT evaluated in Zieglmayer 2016^85^	Primary outcome, and all symptom scores, evaluated in challenge chamber. Symptom scores outside challenge chamber (ie, day-to-day clinical scores) not assessed
Okubo et al, 2017^23^ (Japan: JapicCTI-121848) DFS; DS; STB	90 sites; Japan	Age 12-64 y; moderate-to-severe HDM-induced AR with ≥1-y medication history of AR. AR symptom score ≥7 on ≥7 d of the 14-d run-in period without any symptomatic treatment	Overall: 26.9 ± 11.9	Overall: 20.9% monosensitized to HDM Overall cosensitizations were Japanese cedar pollen, 67%; Japanese cypress pollen, 34%; cat, 26%; orchard grass, 23%; dog, 15%	3 arms, 946 patients; in MRMD arm: 314/281; in Pl arm: 313/286	1x 6 SQ-HDM tablet SL daily; 52 wk of treatment maintenance	Positive outcome: TCRS during the last 8 wk = 3.95 with MRMD vs 5.15 with Pl (*P* = .001, S); relative difference from pL: 23%	Statistical improvement in the following secondary outcomes with MRMD vs with Pl: AR symptom score, conjunctivitis symptom score. No improvement in AR medication score with MRMD vs with Pl. Subanalysis of adolescent population (aged 12-17 y) consistent with overall study results	—
300 IR tablet; Actair; Stallergenes Greer International AG; standardized mix of DP and DF; marketed maintenance dose: 1 × 300 IR tablet SL daily									
Bergmann et al, 2014^29^ (US: NCT00674700; Europe: 2007-001454-77) DFS, DS, STB, LTB	48 sites; 7 countries; Europe	Age 18-50 y; clinical moderate-to-severe HDM-associated AR > 1 y; average ARTSS ≥ 5 (on scale of 0-12)	In MRMD arm: 29 ± 8.5; in Pl arm: 30 ± 9	Monosensitized to HDM: in MRMD arm: 79/153 (52%); in Pl arm: 75/163 (46%)	3 arms, 509 patients; in MRMD arm: 170/133; in Pl arm: 170/141	1 × 300 IR tablet SL daily; 1-y blinded treatment, then 1 y blinded, treatment-free follow-up period	Positive outcome: LS mean of AAdSS at 1 y = 3.18 (MRMD) vs 3.87 (Pl); absolute reduction of –0.69 (*P* = .015, S); relative reduction of –18%	Effect on AAdSS sustained during y 2 primary period (LTB). Statistical improvement in improvement in patient’s global evaluation at 12 mo (with MRMD vs with Pl). Presence of asthma and sensitization status did not affect the efficacy results	—
Demoly et al, 2021^33^ (US: NCT02443805; Europe: 2014-004223-46) DS, STB	231 sites; 13 countries; Europe, USA, Israel	Age ≥ 12 y; HDM-induced AR for ≥ 12 mo prior; self-reported impact on QoL	Overall: 29.6 ± 12.8	Overall 55% monosensitized to HDM Cosensitizations in MRMD arm: cat 141 (20%); grass 131 (19%); birch 93 (13%); ragweed 65 (9%); dog 51 (7%)	2 arms, 1607 patients; in MRMD arm: 802/711; in Pl arm: 805/765	1 × 300 IR tablet SL daily; 12 mo of blinded treatment, then 2 wk of treatment-free follow-up period	Positive outcome: average TCS during 4-wk primary evaluation period at the end of the treatment period LS mean 3.62 with MRMD vs with 4.35 Pl (*P* < .0001, S); relative LS mean difference –17%	Statistical improvement in the following secondary outcomes with MRMD vs with Pl: average RTSS, average RMS, RQLQ12+, 6 individual symptom scores, proportion without rhinitis exacerbation	—
Okamoto et al, 2017^30^ (Japan: JapicCTI-121917) DFS, DS, STB	50 sites; Japan	Age 12-64 y; history of HDM-induced AR for ≥2 y; ARTSS ≥6 of 15 for 7 d before randomization	In MRMD arm: 30.0 ± 11.8; in Pl arm: 30.2 ± 11.6	Monosensitized to HDM: in MRMD arm: 99/315 (31%); in Pl arm: 98/316 (31%)	3 arms, 968 patients; in MRMD arm 322/315; in Pl arm: 323/316	1 × 300 IR tablet SL daily; 52 wk of treatment	Positive outcome: AASS during last 8 wk of the 52 wk of treatment period: 5.00 ± 0.21 with MRMD vs 6.11 ± 0.21 with Pl (*P* = .0001, S); relative LS mean difference –18%	Statistical improvement in the following secondary outcomes with MRMD vs with Pl: average rhinitis total symptom score, average medication score, average combined score, average total rhinoconjunctivitis symptom score, JRQLQ general state	—
Okamoto et al, 2019^31^ (Japan: JapicCTI-152981) DS, STB	Multisite; 51 sites in Japan	Age 5-17 y; AR symptoms for ≥2 y; RTSS ≥ 6 for 7 d before randomization	In MRMD arm: 10.3 ± 2.7; in Pl arm: 10.4 ± 2.7	Monosensitized to HDM: in MRMD arm: 39/205 (19%); in Pl arm: 44/217 (20%)	2 arms, 438 patients; in MRMD arm 219/193; in Pl arm 219/210	1 × 300 IR tablet SL daily; 52 wk of treatment; 1-wk posttreatment observation period	Positive outcome: AASS in the last 4 wk of the 52 wk of treatment period: 6.32 ± 0.20 with MRMD vs 7.27 ± 0.19 with Pl (*P* = .0005); relative LS mean difference –13%	Statistical improvement in the following secondary outcomes with MRMD vs with Pl: average rhinitis total symptom score, average combined score. No improvement in average rescue medication score with MRMD vs with Pl	—
Roux et al, 2016^32^ (US: NCT01527188) DFS, STB	8 sites; Canada	Age 18-55 y; ≥1-y history of HDM-associated AR uncontrolled despite the use of symptomatic treatments; positive EEC result using DP allergen	In MRMD arm: 32.5 ± 8.6; in Pl arm: 31.3 ± 8.8	Monosensitized to HDM: in MRMD arm: 16/86 (19%); in Pl arm: 23/87 (26%)	4 arms, 355 patients; in MRMD arm 86/68; in Pl arm 87/75	1 × 300 IR tablet SL daily; 6 mo of treatment; 2-wk posttreatment observation period	Negative outcome: change from baseline to the end of treatment (6 mo) in the AUC of the RTSS over the 4 h of the challenge with the MRMD vs with Pl: relative LS mean difference –29% (NS)	Mixed results in secondary outcomes for MRMD arm; see article	The study demonstrated a dose-dependent numeric improvement in symptom scores in an environmental challenge chamber, but the improvement in the MRMD dose was not clinically significant vs Pl
SG FDA-standardized extracts; Stallergenes Greer International AG; DF 100%; marketed maintenance dose not specified									
Bush et al, 2011^18^ ES, STB	1 site; United States	Age 18-50 y; ≥ 2-y clinical history of HDM-induced AR. Patients using INCS at baseline excluded	Overall mean: 29.4 y (range nsp)	nsp	3 arms, 31 patients; high-dose arm 10/9; low-dose arm 10/7; Pl 11/5	High-dose arm: 4200 AU SL once daily; low-dose arm: 60 AU SL once daily; 12-18 mo of maintenance treatment	Primary outcome not prespecified	Increased threshold to bronchial allergen challenge in high-dose arm (AgPD20 70 ± 18 cumulative breath units at baseline vs 101 ± 13 cumulative breath units, p = 0.04, S). No significant change in bronchial challenge for low-dose arm vs baseline. Neither high-dose nor low-dose arm showed significant difference in AR symptom and medication scores after 12 mo of treatment	Study was not prospectively registered. Primary outcome was not prespecified. Patients using INCS or OCS excluded. No comparison was made for any outcome between intervention and Pl arms (comparisons were made to baseline period for each arm individually). Outcome of bronchial challenge was reported, but <50% of patients in study carried a diagnosis of asthma, so this is of limited clinical significance. No improvement in AR symptom or medication scores. No power calculation provided; study likely underpowered
Chanllergen; Zhejiang Wolwo Bio-Pharmaceutical Co Ltd, China; DF 100%; marketed maintenance dose: patients ≥ 14 y: 2 drops of 1000-μg/mL solution; patients < 14 y: 3 drops of 333-μg/mL solution									
Chen et al, 2017^74^ ES, STB	No. of sites not stated; China	Age 6-12 y; clinical history of HDM induced AR for >1 y. Patients using oral or nasal corticosteroids excluded	In MRMD arm: 10.1 ± 3.7; in Pl arm: 10.3 ± 3.5	nsp	2 arms, 42 patients; in MRMD arm 21/nsp; in Pl arm 21/nsp	3 drops of 333 mg/mL liquid SL once daily; 12 mo of treatment	Primary outcome not prespecified	Total symptom score averaged over last 12 wk of treatment 5.2 with MRMD vs 7.8 with Pl (*P* < .05, S)	Study was not prospectively registered. Primary outcome was not prespecified. Enrolment criteria did not include an AR severity threshold. AR medication use was not evaluated. Outcomes did not include CSMS
Luo et al, 2014^75^ ES, STB	No. of sites not stated; China	Age 5-16 y; clinical history of HDM-induced AR for ≥2 y	In MRMD arm: mean 9.2 (range 5.1-15.6); in Pl arm: mean 9.5 (range 5.4-16)	100% monosensitized to HDM	2 arms, 72 patients; in MRMD arm 36/nsp; in Pl arm 36/nsp	3 drops of 333 mg/mL liquid SL once daily; 12 mo of treatment	Primary outcome not prespecified	Median SMS score at 12 mo 7.9 with MRMD vs 11.8 with Pl *(P* < .05, S)	Study was not prospectively registered. Primary outcome was not prespecified. Enrolment criteria did not include an AR severity threshold
Wang et al, 2016^76^ ES, STB	No. of sites not stated; China	Age 6-16 y; clinical history of HDM-induced AR for ≥2 y	In MRMD arm: range 6-14; in Pl arm: range 6-16	100% monosensitized to HDM	2 arms, 50 patients; in MRMD arm 25/nsp; in Pl arm 25/nsp	3 drops of 333 mg/mL liquid SL once daily; 12 mo of treatment	Primary outcome not prespecified	TNSS at 12 mo 3.1 ± 0.8 with MRMD vs 7.1 ± 3.3 with Pl (P ≤ 0.05, S). Medication score at 12 mo 5.2 ± 1.4 with MRMD vs 8.3 ± 3.1 with Pl (*P* < .05, S)	Study was not prospectively registered. Primary outcome was not prespecified. Outcomes did not include CSMS, although statistically significant improvement in both symptom score and medication score with Ac vs with Pl was demonstrated
Wen et al, 2018^86^ ES, LTB	No. of sites not stated; China	Age 18-60 y; HDM allergy for ≥ 2 y	In MRMD arm: 34.6 ± 15.1; in Pl arm: 36.8 ± 16.8	nsp	2 arms, 40 patients; in MRMD arm 20/nsp; in Pl arm 20/nsp	2 drops of 1000 mg/mL liquid SL once daily; 24 mo of maintenance treatment	Primary outcome not prespecified	TNSS averaged over 8 wk at 24 mo 1.7 ± 0.8 with MRMD vs 7.7 ± 2.9 with Pl (*P* < .05)	Study was not prospectively registered. Primary outcome was not prespecified. Enrolment criteria did not include an AR severity threshold. AR medication use was not evaluated. Outcomes did not include CSMS
LAIS sublingual tablets; Lofarma SpA, Italy; mix of DP 50% and DF 50%; marketed maintenance dose: 1 × 1000 AU tablet SL 1 to 2 times per wk									
Passalacqua et al, 2006^53^ ES, LTB	Multisite; no. of sites not stated; Italy	Age 18-50 y; ARIA mild persistent rhinitis for ≥2 y	In MRMD arm: 30.6; in Pl arm: 33.6	No. of monosensitized patients not stated; patients with cat or *Parietaria* sensitization excluded from study	2 arms, 68 patients; in MRMD arm 34/28; in Pl arm 34/28	1000 AU sublingual tablet twice weekly for 2 y	Primary outcome not prespecified	Graphical data; mean daily total symptom score with MRMD improved vs with Pl at 12 mo (*P* = .027) but not at 24 mo (*P* NS)	Study was not prospectively registered. Primary outcome not prespecified. Medication and symptom scores evaluated separately; CSMS not reported. Improvement in symptom and medication scores demonstrated at 12 mo but not at 24 mo
SLIMtdc; Inmunotek SL; Spain; allergen composition mixture of DP and DF; marketed maintenance dose: 2 × 100 μL sprays daily SL daily									
Nieto et al, 2022^43^ (US: NCT02661854; Europe: 2015-000820-27) DFS, STB	Multisite; 13 sites; Spain	Age 12-65 y; rhinitis/rhinoconjunctivitis, induced by allergic sensitization to DP and DF	Median (Q1, Q3): In MRMD arm: 25^13^^,^^35^; in Pl arm: 26^17^^,^^41^	Monosensitized to HDM: in MRMD arm: 5/18 (38%); in Pl arm: 10/19 (53%)	9 arms, 196 patients; in MRMD arm 19/18; in Pl arm 20/19	0.2 mL (2 × 0.1 mL sprays) of 3000 Utm/mL of allergen spray daily; 4 mo of maintenance treatment	Positive outcome: NPT after 4 mo of treatment: 61% of subjects receiving MRMD vs 16 % of subjects receiving Pl improved (*P* = .004, S)	AUC of CSMS after 4 mo of treatment 88.5 with MRMD vs 136 with Pl (*P* = .031, S)	This study showed an improvement in NPT and CSMS at the MRMD dose vs with Pl. A phase III study of this dose is under way

*AAdSS*, Average adjusted symptom score; *ARIA*, AR and its Impact on Asthma; *AMS*, asthma medication score; *ASS*, asthma symptom score; *AUC*, Area under the curve; *CPT*, conjunctival provocation test; *DF*, *Dermatophagoides farinae*; *DP*, *Dermatophagoides pteronyssinus*; *EEC*, environmental exposure chamber; *ES*, exploratory studies; *GINA*, Global Initiative for Asthma; *ICS*, inhaled corticosteroid; *INCS*, intranasal corticosteroid; *JRQLQ*, Japan Rhinoconjunctivitis Quality of Life Questionnaire; *LS,* least squares; *LTB*, long-term benefit; *NPT*, nasal provocation test; *NS*, not statistically significant; *nsp*, not specified; *PEF*, peak expiratory flow; *PEFR*, peak expiratory flow rate; *Pl*, placebo; *QoL,* quality of life; *RQLQ*, Rhinitis Quality of Life Questionnaire; *RMS*, rhinitis medication score; *RSS*, rhinitis symptom score; *S*, significant; *sIgE*, specific IgE; *SL*, sublingually; *SMS*, symptom medication score; *STB*, short-term benefit; *TASS*, total asthma symptom score; *TMS*, total medication score; *TRSS*, total rhinitis symptom score; *VAS*, visual analog scale; *VCC*, Vienna Challenge Chamber; *WCA*, well-controlled asthma.

**Table IV:** DBRPCTs of MRMDs of current commercially available SLIT products for HDM-induced AA

Lead author; year of publication; trial registration; classification according to our criteria	Study design; sites	Inclusion criteria: participant age; AA characterization	Population age (y), mean ± SD or median [range])	Sensitization	No arms; total patients R; MRMD: ITT/PP; Pl: ITT/PP	Maintenance dose and schedule; duration of blinded treatment; subsequent unblinded or untreated follow-up	Prespecified primary outcome	Other clinical outcomes of note	Study comments and limitations
12 SQ-HDM tablet; Acarizax (Europe and Australia), Odactra (US), ALK-Abelló A/S, Denmark; standardized mix of DP (50%) and DF (50%); marketed maintenance dose: 1 × 12 SQ-HDM tablet SL daily									
Bozek A et al, 2022 (AA results)^26^ ES; STB	2 sites; Poland	Age ≥ 18 y; diagnosed local AR and concomitant GINA mild-to-moderate chronic asthma; clinical symptoms indicating HDM allergy; positive result of NPT with DP and DF allergens	MRMD: 33.7 ± 3.9 Pl: 35.9 ± 4.2	All patients had positive NPT result to DP and DF, with negative SPT result and sIgE to DP, DF other common aeroallergens	2 arms, 32 patients; MRMD: 17/16; Pl: 15/14	1x 12 SQ-HDM tablet SDLdaily; 12 mo of maintenance	Primary outcome not prespecified	Mean monthly TASS at 12 mo: 0.28 ± 0.27 with MRMD vs 1.28 ± 0.88 with Pl (*P* = .05, S) Mean monthly TMS at 12 mo: 1.07 ± 0.56 with MRMD vs 2.89 ± 2.56 with Pl (*P* = .05, S)	Study was of local AR and AA. Study was not prospectively registered. Primary outcome was not prespecified. AR outcomes did not include CSMS. AA outcome was symptom score only. Outcomes were compared per arm with respect to baseline period, but comparison of MRMD and Pl arms not evaluated
Tanaka A et al, 2020^25^ (Japan: JapicCTI-121847) DFS; DS; STB	124 sites; Japan	Age 18-64 y; AA lasting >6 mo not well controlled by ICS; use of equivalent to fluticasone propionate 200-400 mg at inclusion; FEV1 > 70% predicted; ACQ score between 1 and 1.5	Overall: 38.2 ± 10	Overall, 11% were monosensitized to HDM; 17% were monosensitized to 1 allergen other than HDM; 16% were monosensitized to 2 allergens other than HDM; 56% were monosensitized to ≥3 allergens other than HDM	3 arms; 826 patients MRMD: 277/227 Pl: 275/237	1 × 12 SQ-HDM tablet sublingual daily; 7-13 mo of maintenance treatment, then 6-mo ICS reduction period (period 3)	Negative outcome: time from first randomization to first asthma exacerbation in period 3: 104 d (with MRMD) vs 110 d (with Pl) (*P* = .83, NS)	Prespecified secondary outcomes all negative, see article	Negative study result. Authors postulate reasons for trial failure, including differing definitions of well-controlled asthma between Japanese and GINA guidelines, and different subject selection vs Virchow et al, 2016^24^
Virchow JC et al, 2016^24^ (US: NCT01433523; Europe: 2010-018621-19) DFS; DS; STB	109 sites; Europe	Age ≥ 18 y; AA lasting >1 y not well controlled by ICS; use of equivalent to budesonide 400-1200 μg/d at inclusion; FEV1 > 70% predicted; ACQ score between 1 and 1.5	Overall: 33.4 ± 11.7	Overall, 34% were monosensitized to HDM; 17% were monosensitized to to 1 allergen other than HDM; 15% were monosensitized to 2 allergens other than HDM; 34% were monosensitized to ≥3 allergens other than HDM	3 arms; 834 patients MRMD: 282/248 Pl: 277/257	1 × 12 SQ-HDM tablet sublingual daily; 7-12 mo of maintenance treatment, then 6-mo ICS reduction period (period 3)	Positive outcome: absolute risk of first asthma exacerbation in period 3; 0.24 (with MRMD) vs 0.33 (with Pl); ARR 0.1; *P* = .03, S	No improvement in the following prespecified secondary outcomes: improvement in ACQ score controlled for ICS, improvement in AQLQ(S) score controlled for ICS	Results contradict findings of Tanaka 2020.^25^ Several prespecified secondary outcomes were negative. Authors note that these outcomes used novel assessment methodology
6 SQ-HDM tablet; Miticure (Japan); ALK-Abelló A/S, Denmark; standardized mix of DP (50%) and DF (50%); marketed maintenance dose: 1 × 6 SQ-HDM tablet SL daily									
Mosbech H et al, 2014 (AA results)^27^ (US: NCT00389363; Europe: 2006-001795-20) DFS; DS; STB	81 sites; Europe	Age > 14 y; mild-to-moderate HDM AA; ICS use equivalent to 100-800 mg/d of budesonide for >6 of past 12 mo, FEV1 > 70% predicted; no severe asthma within past 2 y	Graphical data only	Monosensitized to HDM: 28% of entire study cohort	4 arms; 604 patients MRMD: 156/101 Pl: 143/92	1 × 6 SQ-HDM tablet sublingual daily; 1 y of maintenance treatment	Positive outcome: reduction in ICS dose from the baseline dose during 4-wk ICS-stable period: 42% of subjects receiving MRMD vs 15% of subjects receiving Pl (*P* = .0011, S)	Percentage of patients able to discontinue ICS use in 34% of subjects receiving MRMD vs in 21% of subjects receiving Pl, *P* value not stated	Study did not include a 12 SQ-HDM arm, which is the dose used globally outside Japan. Several outcomes reported as numeric comparison between MRMD and Pl arms only, without *P* value
Tanaka A et al, 2020^25^ (Japan: JapicCTI-121847) DFS; DS; STB	124 sites; Japan	Age 18-64 y; AA lasting >6 mo not well controlled; ICS use equivalent to fluticasone propionate 200-400 mg/d at inclusion; FEV1 > 70% predicted; ACQ score between 1 and 1.5	Overall: 38.2 ± 10	Overall, 34% were monosensitized to HDM; 17% were monosensitized to 1 allergen other than HDM; 15% were monosensitized to 2 allergens other than HDM; 34% were monosensitized to ≥3 allergens other than HDM	3 arms; 826 patients MRMD: 274/229 Pl: 275/237	1 × 12 SQ-HDM tablet SL daily; 7-13 mo of maintenance treatment, then 6-mo ICS reduction period (period 3)	Negative outcome: rime from first randomization to first asthma exacerbation in period 3: 104 patients (with MRMD) vs 110 patients (with Pl); *P* = .72, NS	Prespecified secondary outcomes all negative, see article	Negative study result. Authors postulate reasons for trial failure, including differing definitions of well-controlled asthma between Japanese and GINA guidelines, and different subject selection vs Virchow 2016^24^
Virchow JC et al, 2016^24^ (US: NCT01433523; Europe: 2010-018621-19) DFS; DS; STB	109 sites; Europe	Age ≥ 18 y; AA lasting >1 y not well controlled by ICS; ICS use equivalent to budesonide, 400-1200 μg/d at inclusion; FEV1 > 70% predicted; ACQ score between 1 and 1.5	Overall: 33.4 ± 11.7	Overall, 12% were monosensitized to HDM; 14% were monosensitized to 1 allergen other than HDM; 18% were monosensitized to 2 allergens other than HDM; 56% were monosensitized to ≥3 allergens other than HDM	3 arms; 834 patients MRMD: 275/239 Pl: 277/237	1 × 6 SQ-HDM tablet SL daily; 7-to 12-mo maintenance treatment period, then 6-mo ICS reduction period (period 3)	Positive outcome: absolute risk of first asthma exacerbation in period 3; 0.24 (with MRMD) vs 0.33 (with Pl); ARR 0.09; *P* = .045, S	No improvement in the following prespecified secondary outcomes: improvement in ACQ score controlled for ICS, improvement in AQLQ(S) score controlled for ICS	Results contradict Tanaka 2020.^25^ Several prespecified secondary outcomes were negative. Authors note that these outcomes used novel assessment methodology
300 IR tablet; Actair; Stallergenes Greer International AG; standardized mix of DP and DF; marketed maintenance dose: 1 × 300 IR tablet SL daily									
Pham-Thi N et al, 2007^34^ ES; STB	Single site; France	Age 5-15 y; asthma for ≥2 y; use of ICS equivalent to 200-1000 μg/d for > 6 mo of last 12 mo; reversible bronchial obstruction; FEV1 > 15% predicted	MRMD: 9.6 (5-14) Pl: 9.5 (5-16)	Patients were sensitized to seasonal and perennial allergens that could confound assessments excluded	2 arms, 111 patients; MRMD: 55/54; Pl: 56/55	1 × 300 IR tablet SL daily; 17.5 mo of maintenance treatment	Negative outcome: diurnal asthma symptom score after 18 mo of treatment: 0.15 ± 0.26 with MRMD vs 0.08 ± 0.17 with Pl (NS)	Prespecified secondary outcomes all negative, see article	Study was negative from standpoint of its primary outcome. The authors suggested that the patients had insufficiently severe asthma to detect a clinically meaningful difference between the MRMD and Pl groups
300 IR/mL SLIT spray; Staloral; Stallergenes Greer International AG; mix DP 50% and DF 50%; marketed maintenance dose: 300 IR daily (5 × 200 μg of pressures of 300 IR/mL formulation) daily									
Niu C et al, 2006^71^ ES; STB	5 sites; Taiwan	Age 6-12 y; mild-to-moderate (GINA steps II-III) asthma for >1 y; FEV1 > 70% predicted; PEFR increase > 15% after bronchodilators	MRMD: 7.9 ± 1.6 y; Pl: 8.2 ± 1.7 y	All patients were monosensitized to HDM	2 arms; 110 patients; MRMD: 56/49; Pl 54/48	300 IR/d (20 drops of 300 IR/mL solution daily; the manufacturer confirmed dose of 50 μL/dropper used; see Table E2)	Primary outcome not prespecified	MRMD arm showed improvement in DSS of –0.07 (0.11 at baseline to 0.04 at 24 wk) whereas Pl arm showed a slight worsening of +0.01 (0.05 at baseline to 0.06 at 24 wk). These results were statistically significant (*P* = .028). However, the groups were not well balanced at baseline (DSS 0.11 MRMD vs 0.05 Pl). Further, no improvement was seen in objective outcomes, including PEF and lung function test metrics. Neither a reduction in ICS dose nor reduction of asthma flares was evaluated	Study was not prospectively registered. Primary outcome not prespecified. The study groups were not well balanced at baseline; the MRMD arm had a significantly higher daily symptom score. Improvement seen only in daily symptom score, a patient-reported outcome, not in objectively measured outcomes such as lung function test metrics
Wang L et al, 2014^37^ (US: NCT00660452) DS; STB	14 sites; China	Age > 14-50 y; mild or moderate, persistent, HDM-induced asthma for ≥12 mo	MRMD: 31 (14-50); Pl: 31 (16-49)	Patients were sensitized to seasonal and perennial allergens that could confound assessments excluded	2 arms; 484 patients; MRMD: 322/308; Pl 162/157	300 IR/d; 12-wk baseline phase; 52 wk of treatment; ICS stepdown phase in wk 24 to wk 40; efficacy measured at wk 32 to wk 52	Negative outcome: percentage with WCA at 12-mo od MRMD vs Pl not different (*P* = .244, NS)	*Post hoc* analysis of patients moderately severe asthma showed improvement in WCA in MRMD arm vs in Pl arm (80.5% vs 66.1%; *P*.021, S)	The primary outcome of percentage of patients with well-controlled asthma was not greater with MRMD than with Pl. An improvement with MRMD vs Pl was found only in a *post hoc* subgroup of those with moderate asthma only
Chanllergen; Zhejiang Wolwo Bio-Pharmaceutical Co Ltd, China; DF 100%; marketed maintenance dose: patients ≥ 14 y: 2 drops of 1000-μg/mL solution; patients < 14 y: 3 drops of 333-μg/mL solution									
Tian M et al, 2014^77^ ES; STB	Single site; China	Age 4-18 y; mild-to-moderate AA ± AR	MRMD: 11.1 ± 3.9 Pl: 10.8 ± 3.8	Not stated	2 arms, 60 patients; MRMD:30/nsp; Pl: 30/nsp	3 × 333 μg/mL of drops SL once per d (40 μL per drop); 44 wk of maintenance treatment	Primary outcome not prespecified	Mean daily symptom score at 48 wk: 0.16 ± 0.08 for patients receiving MRMD vs 1.4 for patients receiving Pl (*P* < .05)	Study was not prospectively registered. Primary outcome was not prespecified. Enrolment criteria did not include an AR severity threshold. AR medication use was not evaluated. CSMS was not evaluated

*ACQ*, Asthma Control Questionnaire; *ARR*, absolute risk reduction; *AQLQ(S),* Asthma Quality of Life Questionnaire (Standardized); *CSMS*, combined symptom medication score; *DF*, *Dermatophagoides farinae*; *DP*, *Dermatophagoides pteronyssinus*; *DSS,* daily symptom score*; ES*, exploratory study; *GINA*, Global Initiative for Asthma; *ICS*, inhaled corticosteroid; *NPT*, nasal provocation test; *NS*, not statistically significant; *nsp*, not specified; *PEF*, peak expiratory flow; *PEFR*, peak expiratory flow rate; *Pl*, placebo; *sIgE*, specific IgE; *SL*, sublingually; *SPT*, skin prick test; *STB*, short-term benefit; *TASS*, total asthma symptom score; *TMS*, total medication score; *WCA*, well-controlled asthma.

**Table V:** DBRPCTs of MRMDs of current commercially available SCIT products for HDM-induced AR

Lead author; year of publication; trial registration; classification according to our criteria	Study design; sites	Inclusion criteria: participant age; AR characterization	Population age (y; mean ± SD or median [range])	Sensitization	No arms; total patients R; MRMD: ITT/PP; Pl: ITT/PP	Maintenance dose and schedule; duration of blinded treatment; subsequent unblinded or untreated follow-up	Prespecified primary outcome	Other clinical outcomes of note	Study comments and limitations
Acaroid; Allergopharma GmbH & Co KG; Germany; allergen composition DP 100%; marketed maintenance dose: 6,000 TU every 4-8 wk									
Unpublished; trial completed 2010 (US: NCT00540631^87^; Europe: 2006-000934-11^78^; company study ID: AL0106ac) ES; LTB	31 sites; Germany, Poland and Austria	Moderate-to-severe AR triggered by HDM; positive CPT result ; AUC of SMS (last 4 wk of 3-month baseline period) ≥ 84 and < 700	MRMD: median 27 (range nsp); Pl: median 27 (range nsp)	Patients sensitized to seasonal and perennial allergens that could confound assessments excluded	2 arms; 108 patients; MRMD: 51/47; Pl: 57/50	6000 TU every 4-6 wk for 2 y	Negative outcome: change of AUC of SMS after 2 y of treatment MRMD vs Pl not different (*P* = .7002, NS)	Prespecified secondary outcome of AUC of SMS not improved MRMD vs Pl. Percentage of patients with 40% decrease in AUC of SMS was numerically worse with MRMD vs with Pl (46.8% vs 60%, *P* nsp)	Study result was considered negative. Trial results published only to European Clinical Trials Registry; results not published in peer-reviewed literature
Dokic D et al, 2005^38^ ES; LTB	Single site; Macedonia	Age ≥ 18 y; history of AR due to HDM; symptom severity on VAS v4-10; positive DP NPT result	MRMD: mean 27 (18-57 range); Pl: mean 33 (18-62 range)	nsp	2 arms; 40 patients MRMD: 20/20 Pl: 20/20	6000 TU every 4-8 wk; 2-y blinded treatment followed by further 1 y of unblinded treatment (MRMD arm only)	Negative outcome: patient VAS not significantly different between groups (numeric values nsp; *P* value NS)	Improvement in nasal challenge scores at 24 mo improved with MRMD vs with Pl (values not stated; *P* < .05, S)	Study was not prospectively registered. 1 site did not recruit patients; total enrolment was planned at 80 patients but only 40 enrolled. Primary outcomes not prespecified. CSMS was not evaluated. Mixture of positive and negative outcomes reported across multiple end points. Outcomes described as statistically significant but clinical significance of outcomes not established
ALK-depot SQ 503 *Dermatophagoides pteronyssinus* (Alutard SQ); ALK-Abelló A/S, Denmark; allergen composition DP 100%; marketed maintenance dose: 100,000 SQ-U every 4-8 wk									
Varney V A et al, 2003^79^ ES; STB	2 sites; England	Age > 18 y; troublesome clinical symptoms of moderate-to-severe HDM-induced perennial AR despite standard therapy and avoidance measures	MRMD: mean 33 (range 19-48) Pl: mean 37 (range 23-55)	nsp	2 arms, 36 patients; MRMD: 19/15; Pl: 17/13	1 × 100,000 SQ-U injection monthly; 1 y of maintenance treatment	Primary outcome not prespecified	Total symptom score at 1 y: 72 ± 26 with MRMD vs 132 ± 48 with Pl (NS)	Symptom and medication score not significantly different between Ac and Pl groups at end of study. Study was not prospectively registered. Primary outcome was not prespecified. CSMS was not evaluated. Study likely underpowered
Xian M et al, 2020^80^ ES; STB	Single site; China	Age 5-55 y; ARIA mild-severe AR	MRMD: 21 ± 12 Pl: 25 ± 6	Patients sensitized to seasonal and perennial allergens that could confound assessments excluded	3 arms, 70 patients; MRMD: 28/26; Pl 14/14	1 × dose of 100,000 SQ-U injection every 6 wk; 12 mo of treatment	Primary outcome not prespecified	TRS at 12 mo: 3.44 ± 2.82 with MRMD vs 6.7 ± 2.8 with Pl (*P* < .05, S)	Study was not prospectively registered. Primary outcome was not prespecified. Enrolment criteria did not include an AR severity threshold
Allergovac; Roxall Medizin GmbH; Germany; allergen composition DP 100%; marketed maintenance dose: 1000 TSU every 4 wk									
Moreno V et al, 2016^39^ (US: NCT01564017; Europe: 2011-004583-30) DFS; STB	12 sites; Spain	Age 18 60 y; perennial allergic rhinoconjunctivitis due to DP sensitization for ≥2 y	MRMD: 27 ± 9 Pl: 28 ± 8	Patients sensitized to seasonal and perennial allergens that could confound assessments excluded	6 arms, 136 patients; MRMD: 17/13; Pl: 20/18	1 × 1000 TSU (0.125 SPT) every 4 wk; 12 wk of maintenance treatment	Negative outcome in MRMD arm: change in the concentration of DP extract needed to produce a positive NPT result at the final visit compared with baseline: 0.8 MRMD vs 0.3 Pl (both groups NS relative to baseline)	—	The MRMD arm did not show statistical improvement at final visit vs baseline. The only clinical outcome reported was allergen threshold for positive NPT result; symptom and medication score use was not assessed. Comparisons made for each arm between baseline period and final visit; no statistical comparison between arms at final visit. Study likely underpowered
Depigoid; LETI Pharma, SLU; Spain; allergen composition mixture of DP and DF; marketed maintenance dose: 50 DPP every 30 d									
Garcia-Robaina J et al, 2006^82^ (AR results) ES; STB	Single site; Spain	Eligibility age range not stated (results imply adults and adolescents included); ARIA moderate-to-severe AR due to DP and DF	MRMD: 24 ± 9 Pl: 24 ± 8	Patients with sensitization to other common allergens were excluded	2 arms, 64 patients; MRMD: 32/27; Pl: 32/27	1 × 0.5 mL of 35μg/mL DP + 28 μg of DF injection every month; 12 mo of maintenance treatment; the manufacturer confirmed that this is equal to MRMD of 50 DPP/mo; see Table E2	Primary outcome not prespecified	Total symptom score at 12 mo = 11.63 for subjects receiving MRMD vs 25.12 for subjects receiving Pl (*P* < .001, S). Total medication score at 12 mo = 10.47 for subjects receiving MRMD vs 22.09 for subjects receiving Pl (*P* < .001, S)	Study was not prospectively registered. Clinical outcomes were BPT result, asthma symptom score and asthma medication score; neither reduction in ICS use nor reduction of asthma flares was evaluated
Novo-Helisen Depot; Allergopharma GmbH & Co KG; Germany; allergen composition DP 100%; marketed maintenance dose: 5000 TU every 4-6 wk									
Unpublished; trial completed 2006 (results obtained from study sponsor) (US: NCT00263549 (83) ; company study ID: Al0201NH) ES; LTB	11 centers; Germany and The Netherlands	Eligibility age range not stated; patients included aged 18-56 y; AR/C due to HDM; positive DP CPT result	MRMD: mean 33 (range 18-56) Pl: mean 32 (range 18-56)	Patients with other perennial allergies excluded	2 arms; 135 patients; MRMD: 70/nsp Pl: 65/nsp (total 64 patients in PP set)	5000 TU every 5 ± 1 wk for 2 y	Primary outcome not prespecified	In PP set, reduction in SMS at 2 y vs at baseline seen for 88% of subjects receiving MRMD vs for 36% of subjects receiving pL (*P* = .017, S). Results of ITT population nsp	Study was not prospectively registered; although study is listed in the US Clinical Trials Registry, date of listing (December 2005) is after the study was completed according to the poster (February 2005). Study outcomes not published either to clinical trials registry or in peer-reviewed literature; outcomes were published as a conference poster which was obtained from the AIT manufacturer. Primary outcome not prespecified. Symptom/medication score improved only in per-protocol analysis; no improvement in ITT population
Novo-Helisen Depot; Allergopharma GmbH & Co KG; Germany; allergen composition mixture of DP 50% and DF 50%; marketed maintenance dose: 5000 TU every 4-6 wk									
Yukselen A et al . 2012^84^ (AR results) ES; STB	Single site; Turkey	Children; eligibility age range not stated. Clinical history of ≥1 y of AR with asthma related to HDM symptoms and no previous AIT	MRMD: 11 ± 3 Pl: 10 ± 3	All participants monosensitized to HDM	3 arms, 32 patients; MRMD: 10/nsp; Pl 10/nsp	Induction phase involved escalation to maximum of 4000 TU/injection (0.8 mL of 5000 TU/mL formulation). The maximum dose tolerated during the induction phase was the maintenance dose, which was given every 4 wk. Mean cumulative SCIT dose over 12 mo = 43770 TU, including in induction period; average maintenance dose not stated	Primary outcome not prespecified	RSS lower in MRMD group than Pl at 12 mo (*P* = .03, S). RMS lower in MRMD group than in Pl group at 12 mo (*P* = .05, S)	Only 10 patients in SCIT arm. Study was not prospectively registered. Primary outcome not prespecified
Purethal Mites; HAL Allergy BV, The Netherlands; allergen composition mixture of DP 50% and DF 50%; marketed maintenance dose: 10,000 AUeq every 4 wk									
Bozek A et al, 2017^40^ (US: NCT03209245; company study ID: HDMeld) ES; LTB	Single site; Poland	Age > 65 y; ARIA moderate or severe intermittent AR; positive result of NPT to DP and DF allergens	MRMD: 68.1 ± 5.9 Pl: 69.2 ± 6.3	All participants monosensitized to HDM	2 arms, 58 patients; MRMD: 30/29; Pl: 28/26	1 × 0.5 mL injection of 20,000-AUeq/mL dose every 4 wk; 24 mo of maintenance treatment	Positive outcome: after 2 y of treatment, AAdSS of 1.82 ± 0.71 in MRMD arm vs 3.97 ± 0.96 in Pl arm (*P* < .05, S)	RQLQ at 24 mo improved in MRMD arm but not in Pl arm	—
Pfaar O et al, 2016^45^ (US: NCT01438463; Europe: 2011-000393-61) DFS; STB	Multisite; 34 sites; Europe	Age 18-60 y; HDM-induced ARC for ≥1 y; positive result of NPT to HDM extract	MRMD: 32 ± 10 Pl: 30 ± 10	Patients with clinically relevant ARC due to allergens other than HDM were excluded	5 arms; 290 patients; MRMD: 59/59; Pl: 56/56	10,000 AUeq (0.5 mL of 20,000 AUeq formulation) every 4 wk; total study duration was 12 mo; average of 9.5 maintenance doses per patient	Positive outcome for MRMD arm: 42% reduction in AAdSS following NPT at 12 mo for MRMD arm vs for Pl arm (*P* = .04, S)	AAdSS score in MRMD arm for last 8 wk of treatment not improved vs in Pl arm	This study showed improvement in symptom score during NPT at the MRMD vs with Pl but did not show improvement based on symptom scores during the last 8 wk of the treatment period. The subsequent DS of Purethal Mites was performed using a higher dose than the current MRMD (0.5 mL of 50,000 AUeq/mL vs 0.5 mL of 20,000 AUeq/mL). Results of this study were published to the European Clinical Trials Registry^51^ but not in the peer-reviewed literature

*AAdSS*, Average adjusted symptom score; *ARIA*, Allergic Rhinitis and its Impact on Asthma; *AMS*, asthma medication score; *BPT*, bronchial provocation test; *CPT*, conjunctival provocation test; *DF*, *Dermatophagoides farinae*; *DP*, *Dermatophagoides pteronyssinus*; *ES*, exploratory studies; *ICS*, inhaled corticosteroid; *LTB*, long-term benefit; *NPT*, nasal provocation test; *NS*, not statistically significant; *nsp*, not specified; *Pl*, placebo; *RMS*, rhinitis medication score; *RSS*, rhinitis symptom score; *RQLQ*, Rhinitis Quality of Life Questionnaire; *SMS*, symptom medication score; *STB*, short-term benefit; *TMS*, total medication score; *TRS*, total rhinitis score; *VAS*, visual analog scale.

**Table VI:** DBRPCTs of MRMDs of commercially available SCIT products for HDM-induced AA

Lead author; year of publication; trial registration; classification according to our criteria	Study design; sites	Inclusion criteria: participant age; AA characterization	Population age (y; mean ± SD or median [range])	Sensitization	No arms; total patients R; MRMD: ITT/PP; Pl: ITT/PP	Maintenance dose and schedule; duration of blinded treatment; subsequent unblinded or untreated follow-up	Prespecified primary outcome	Other clinical outcomes of note	Study comments and limitations
Acaroid; Allergopharma GmbH & Co KG; Germany; allergen composition DP 100%; marketed maintenance dose: 6,000 TU every 4-8 wk									
Unpublished; trial completed 2009 (adult results only; see Table E2) (US: NCT00263640^88^; Europe: 2004-003892-35^46^; company study ID: Al0104av) ES; LTB	Multisite; no. of sites not stated; Germany and Poland	Age 18-64 y; mild-to-moderate HDM AA (GINA II-III); requirement of ICS equivalent to 100 mg of fluticasone per d; positive result of CPT with DP allergens	Eligibility age range 18-64 y; no further data available	Patients with clinically relevant symptoms of AR due to other allergens during assessment period were excluded	2 arms; 64 patients; MRMD: 30/27; Pl: 34/29	1 × 6000 TU (or maximum tolerated dose) injection every 6 wk; 2 y of maintenance treatment	Negative outcome in adults: regarding changes from baseline in fluticasone dose steps after 2 y of treatment, no difference with MRMD vs with Pl (*P* = .4533, NS)	No improvement in key secondary outcomes with MRMD vs with Pl in adults	Study in adults was Pl-controlled and results were considered negative. Study in children was open-label, and results were considered positive. Only the results in children have been published in the peer-reviewed literature^89^; results in adults are available only from the European trials registry
Jutel M et al, 2018^41^ (Europe: 2011-002248-29; company study ID: AL1009ac) DFS; LTB	36 sites; Poland, Spain	Age 18-40 y; allergic bronchial asthma caused by HDM sensitization; asthma controlled (GINA); history of ICS treatment with maximum fluticasone equivalent of 500 μg/d	MRMD: 28 ± 7 Pl: 28 ± 8	Patients with clinically relevant symptoms of AR due to other allergens during assessment period were excluded	5 arms, 146 patients; MRMD: 31/29; Pl: 32/29	1 × 6000 TU injection every 4 wk; 19 wk of maintenance treatment	Positive outcome according to primary end point; however, primary outcome was not clinically significant: reduction in swelling area of late-phase reaction at injection site (6.3 cm^2^ with MRMD vs -6.41 cm^2^ with Pl, stated to be significant, but *P* value NS)	Change in minimum ICS dose for asthma control not significant with MRMD vs with Pl (subset analysis of patients without asthma control at baseline) Asthma control test with MRMD (LS mean ± SE), 4.83 ± 1.22, n = 9 vs with Pl 1.77 ± 1.08, n = 13; *P* = .0432, S Average morning PEF not significantly improved in MRMD vs Pl	Primary outcome was not clinically relevant (change of swelling size at injection site). Clinical outcomes analyzed in subgroup that needed an ICS at enrolment (73 patients[ 46% of study population]); whether this was a prespecified or *post hoc* analysis is not specified. The main clinical outcome of ICS dose required for asthma control was not improved in the MRMD arm (6000 TU/d); it was positive only in the 18,000-TU/d arm (which is 3 times the current MRMD)
ALK-depot SQ 503 *Dermatophagoides pteronyssinus* (Alutard SQ); ALK-Abelló A/S, Denmark; allergen composition DP 100%; marketed maintenance dose: 100,000 SQ-U every 4-8 wk									
Blumberga G et al, 2006^42^ ES; LTB	Single site; Germany	Age 18-60 y; perennial asthma requiring long-term ICS equivalent to FP at dos of 500-2000/μg daily; FEV1 > 70% predicted	MRMD: 29.8 ± 10.7; Pl: 28.5 ± 7.1	100% DP, 72% grass, 65% dog, 52% cat, 35% birch pollen Patients sensitized to cat or dog excluded if daily contact with pet. Patients sensitized to *Cladosporium herbarium* and *Alternaria alternata* were excluded	2 arms; 54 patients MRMD: 26/20 Pl: 28/25	100,000 SQ-U every 6 ± 2 wk; 3 y	Negative outcome: ICS dose reduction at 3 y: 82% (MRMD) vs 42% (Pl); *P* = .17, NS	*Post hoc* analysis of patients with moderate asthma (use of 500 to 1000 of μg FP daily at baseline): statistically significant reduction in ICS dose was found after 2 y (*P* = .01, S) and 3 y (*P* = .04, S)	Prespecified primary outcome was negative; several *post hoc* secondary outcomes were positive. Study was not prospectively registered
Wang H et al, 2006^81^ ES; STB	3 sites; China	Age 6-45 y; GINA mild-to-moderate asthma; FEV_1_ < 60% predicted; maximum of 500 μg/d of budesonide for last 3 mo	Subjects aged <16 y: MRMD: 10 ± 0.4 Pl: 11 ± 0.4 Subjects aged >16 y: MRMD: 32 ± 2 Pl: 29 ± 2	Monosensitized to HDM: for MRMD arm, 38/64 (59%) vs for P arm: 41/65 (63%). Patients sensitized to pets at home were excluded	2 arms, 132 patients; MRMD: 66/64; Pl: 66/65	26-wk initiation period to maximum dose of 100,000 SQ-U, then given as maintenance every 6 wk; 27 wk of maintenance treatment	Primary outcome not prespecified	Mean daily symptom score: 0.171 ± 0.041 with MRMD vs 0.390 ± 0.093 with Pl (*P* = .032, S) Mean daily medication score: significantly lower with MRMD than with Pl by end of treatment (*P* = .016, S)	Study was not prospectively registered. Primary outcome was not prespecified. Improvement was demonstrated only in subjective assessments such as asthma symptoms score; objective scores, including no. of asthma flares and peak flow values, were not significantly different for Ac vs for Pl
Depigoid; LETI Pharma, SLU; Spain; allergen composition mixture of DP and DF; marketed maintenance dose: 50 DPP every 30 d									
Garcia-Robaina J et al, 2006^82^ (AA results) ES; STB	Single site; Spain	Eligible age range not stated (results imply that adults and adolescents are included); GINA mild-to-moderate asthma due to HDM	MRMD: 24 ± 9 Pl: 24 ± 8	Patients sensitized to common allergens other than DP and DF excluded	2 arms, 64 patients; MRMD: 32/27; Pl: 32/27	1 × 0.5 mL of 35 μg/mL of DP + 28 μg of DF injection every month; the manufacturer confirmed that this is equal to MRMD of 50 DPP/mo; see Table E2; 12 mo of maintenance treatment	Primary outcome not prespecified	Improvement in BPT result at 12 mo in MRMD arm (*P* < .001, S), but not in Pl arm (*P* = .6, NS) No. of patients who needed more than twice the amount of allergen to obtain a positive BPT result: 20 with MRMD vs 9 with Pl (*P* = .013, S)	Study was not prospectively registered. Clinical outcomes were BPT result, asthma symptom score and asthma medication score; neither reduction in ICS use nor reduction of asthma flares was evaluated
Depigoid; LETI Pharma, S.L.U.; Spain; allergen composition DP 100%; marketed maintenance dose: 50 DPP every 30 d									
Ameal A et al, 2005^49^ ES; STB	Single site; Spain	Eligibility age not stated; patients aged 14-48 y enrolled; GINA mild-to-moderate asthma; positive BPT result	Mean age across both arms: 23 y (range 14-48 y) No statistical difference in age between 2 arms	Patients sensitized to common allergens other than DP and DF excluded	2 arms, 63 patients; MRMD: 32/29; Pl: 31/26	0.5 mL of 70-μg/mL extract per month, subcutaneous injection; the manufacturer confirmed that this is equal to MRMD of 50 DPP/mo; see Table E2; 12 mo of maintenance treatment	Primary outcome not prespecified	Improvement in BPT threshold dose at 12 mo improved in MRMD group vs in Pl group (P= .0029, S). At 12 mo, symptom score with MRMD was 0.14 (IQR: 0.00-1.93) vs 2.93 (IQR: 1.11-4.19) with Pl (*P* = .0001, S). At 12 mo, medication score was 1.00 (IQR: 0.29-1.50) with MRDM vs 3.13 (IQR: 2.473.63) with Pl (*P* < .0001, S)	Study was not prospectively registered. Clinical outcomes were BPT result, asthma symptom score and asthma medication score; neither reduction in ICS use nor reduction of asthma flares was evaluated
Novo-Helisen Depot; Allergopharma GmbH & Co KG; Germany; allergen composition mixture of DP 50% and DF 50%; marketed maintenance dose: 5000 TU every 4-6 wk									
Yukselen A et al, 2012^84^ (AA results) ES; STB	Single site; Turkey	Children; eligibility age range not stated. Clinical history of ≥1 y of AR with asthma related to HDM symptoms and no previous AIT	MRMD: 11 ± 3 Pl: 10 ± 3	All participants monosensitized to HDM	3 arms, 32 patients; MRMD: 10/nsp; Pl 10/nsp	Induction phase involved escalation to maximum of 4000 TU/injection (0.8 mL of 5000 TU/mL formulation). The maximum dose tolerated during the induction phase was the maintenance dose, which was given every 4 wk. Mean cumulative SCIT dose over 12 mo = 43770 TU, including induction period; average maintenance dose not stated	Primary outcome not prespecified	ASS lower in MRMD group than in Pl group at 12 mo (*P* = .01, S). AMS lower in MRMD group than in Pl group at 12 mo (*P* = .05, S)	Only 10 patients in SCIT arm. Study was not prospectively registered. Primary outcome not prespecified

*ASS*, asthma symptom score; *BPT*, bronchial provocation test; *CPT*, conjunctival provocation test; *DF*, *Dermatophagoides farinae*; *DP*, *Dermatophagoides pteronyssinus*; *DU*, development unit; *ES*, exploratory studies; *FP*, fluticasone propionate; *GINA*, Global Initiative for Asthma; *HR*, hazard ratio; *ICS*, inhaled corticosteroid; *IQR,* interquartile range; *LTB*, long-term benefit; *NS*, not statistically significant; *nsp*, not specified; *PEF*, peak expiratory flow; *Pl*, placebo; *STB*, short-term benefit; *TU*, therapeutic unit.

### Studies with DFSs and DFs

There were 2 products, namely, the ALK 12 SQ-HDM and ALK 6 SQ-HDM SLIT tablets and the SG 300 IR SLIT tablet, that had a paired DFS and DS set, hence demonstrating the efficacy of the current MRMD of the AIT product.

### The ALK 12 SQ-HDM SLIT tablet and 6 SQ-HDM SLIT tablet

For the ALK 12 SQ-HDM SLIT tablet (marketed as Acarizax in Europe and Australia but as Odactra in the United States) and the ALK 6 SQ-HDM SLIT tablet (marketed as Miticure in Japan), we identified a total of 9 studies (in which a total of 6298 patients were randomized). Of these studies (examining a total of 4002 randomized patients), 5 were for the treatment of AR,^19^, ^20^, ^21^, ^22^, ^23^ 2 (with a total of 1660 patients randomized) examined AA,^24^^,^^25^ and 2 (with a total of 636 patients randomized) examined both AA and asthma.^26^^,^^27^ Studies conducted in Japan^20^^,^^23^^,^^25^ describe the dose in Japanese allergy units, with 10,000 Japanese allergy units equating to a dose of 6 SQ-HDM tablets. All of the identified studies included 1 or more MRMD arms (ie, a 6 SQ-HDM arm,^20^^,^^27^ 12 SQ-HDM arm,^22^^,^^26^ or both^19^^,^^21^^,^^23^, ^24^, ^25^).

With regard to trials examining AR (including the trials investigating both AR and AA), we identified a total of 7 studies: 1 DFS,^21^ 2 DSs,^20^^,^^22^ 3 studies that met our criteria for both DFSs and DSs,^19^^,^^23^^,^^27^ and 1 ES.^26^ The studies enrolled a total of 4638 patients; 1432 patients in 5 trials received the 12 SQ-HDM dose,^19^^,^^22^^,^^23^^,^^26^ and 1073 patients in 5 trials received the 6 SQ-HDM dose.^19^, ^20^, ^21^^,^^23^^,^^27^ Six studies prespecified their primary outcome; in all of these studies, the primary outcome was positive.^19^, ^20^, ^21^, ^22^, ^23^^,^^28^

For AA, we identified 4 studies: 2 DSs investigating both the 12 SQ-HDM and 6 SQ-HDM doses,^24^^,^^25^ 1 DFS that included a 6 SQ-HDM dose arm but not a 12 SQ-HDM dose arm,^27^ and 1 ES that included a 12 SQ-HDM arm but not a 6 SQ-HDM arm.^26^ Three studies prespecified their primary outcome; in 2 studies the primary outcome was positive,^24^^,^^27^ and in 1 study it was negative.^25^

### The SG 300 IR SLIT tablet (Actair and Orylmyte)

For the SG 300 index of reactivity (IR) SLIT tablet (marketed as Orylmyte in Germany and Actair elsewhere), we identified 5 studies of the MRMD (300 IR daily dose) in AR.^29^, ^30^, ^31^, ^32^, ^33^ The 5 studies of the 300 IR daily dose randomized a total of 3877 patients, of whom 1599 were treated with the 300 IR daily dose. We identified 1 DFS,^32^ 2 DSs,^31^^,^^33^ and 2 studies that met our criteria for both a DFS and a DS.^29^^,^^30^ All 5 studies prespecified their primary outcome; in 4 studies the primary outcome was positive,^29^, ^30^, ^31^^,^^33^ and in 1 study it was negative.^32^

In AA, we identified 1 ES of the MRMD,^34^ which was reported as negative according to its primary outcome, as well as 2 studies that investigated doses other than the MRMD.^35^^,^^36^

### Other products

For the remaining products, we found a mixture of DFSs, DSs, and ESs; we did not find a paired DFS and DS set demonstrating the efficacy of the current MRMD of any of these products. Notably, we identified only 1 study of the FDA-standardized allergen extracts,^18^ a small ES that randomized a total of 31 patients. Across these products, 10 studies prespecified their primary outcome,^37^, ^38^, ^39^, ^40^, ^41^, ^42^, ^43^, ^44^, ^45^, ^46^ and of these 10 studies, 4 were positive according to their prespecified primary outcome.^40^^,^^41^^,^^43^^,^^45^ The studies identified are summarized in Table II, Table III, Table IV, Table V, Table VI and E3.

## Discussion

AIT product selection must be individualized to each patient and must consider multiple factors, including evidence, compliance, cost, and convenience.^47^ We have performed the first systematic review of DBRPCTs of HDM AIT for the treatment of AA and AR that excludes studies of non–commercially available products and groups studies by the AIT product used and the AIT dose use (MRMD vs other). As such, to our knowledge, ours is the first article that allows allergists in a particular country to compare the evidence for the products that are available to them.

The results of our study are notable for several reasons. First, of the 105 DBRPCTs identified, 30 were trials of products that are no longer commercially available and 8 were trials of products that had never been commercially marketed. These studies, therefore, cannot be used to guide AIT product selection and should be regarded as of historic interest only. The oldest study meeting our inclusion criteria, a study of an allergoid SLIT product, was published in 1998.^48^ The oldest SCIT studies that met our inclusion criteria were published in 2005.^38^^,^^49^ Thus, all of the double-blind, randomized placebo-controlled studies of HDM SLIT published before 1998 and all of the SCIT studies published before 2005 are of products that are no longer commercially available or were never commercialized and are thus of limited relevance to an allergist working in clinical practice today.

Second, we have shown that only 3 products, the ALK 12 SQ-HDM SLIT tablet, the ALK 6 SQ-HDM tablet, and the SG 300 IR SLIT tablet, have a matched DFS and DS pair demonstrating the efficacy of the MRMD of the AIT product for AR, and 2 products (ie, the ALK 12 SQ-HDM SLIT tablet and the ALK 6 SQ-HDM tablet) have a matched DFS and DS pair for the MRMD dose in AA.

Third, our work demonstrates the relatively small evidence base for SCIT versus for SLIT. The total number of patients enrolled in all of the SLIT trials (N = 12,418 patients) was 6 times the total number enrolled in the SCIT trials (N = 2,080). Furthermore, no SCIT product has a DFS and DS pair supporting the MRMD of the product.

Fourth, our work allows comparison of the size of the evidence base for individual products. Notably, the number of patients enrolled in studies of the 6 SQ-HDM SLIT and 12 SQ-HDM SLIT tablets (N = 6298) is 3 times the total number of patients randomized in all studies of other currently commercially available SCIT products combined (N = 2080). A single study of the SG 300 IR SLIT tablet in AR enrolled 1607 patients,^33^ which is nearly as many as the total number of patients enrolled in all studies of commercially available SCIT products for the treatment of AR (N = 1621 patients).

Fifth, our analysis of the dose used in each study highlights several products for which the current MRMD is not the dose for which the best evidence is available. For the Allergopharma allergoid SCIT (Acaroid), the only DS identified used an AIT dose that is 3 times greater than the current MRMD (18,000 therapeutic units vs 6,000 therapeutic units).^50^ Similarly, for the HAL Allergy allergoid SCIT (Purethal Mites), the only DS identified used 2.5 times the current MRMD (25,000 allergenic units [AU]/mL vs 10,000 AU/mL).^51^ Notably, although the outcomes for both trials have been published to the European Clinical Trials Registry, neither trial has been published in peer-reviewed scientific literature, and the MRMD has not been updated to match the dose used in the DS for either product. For the Lofarma SLIT tablet (Lais Mites), a DFS using more than 5 times the MRMD (3000 AU 7 days per week vs the MRMD of 2000 AU twice weekly according to the Lais product information document) did not show a statistically significant benefit versus placebo.^52^ Thus, although 2 small ESs of the Lofarma SLIT tablet have been published,^48^^,^^53^ the studies taken together do not support the clinical efficacy of the Lofarma SLIT tablet at any dose.

Finally, our study highlights the paucity of evidence for the FDA-standardized allergen extracts, for which we identified only a single ES that randomized 31 patients and showed no improvement in AR symptom or medication scores. Notably, only 2 of the products included in this review are available in the United States: the FDA-standardized allergen extracts and the 12 SQ HDM tablet, which are marketed in the United States as Odactra.

This study included only products for which at least 1 DBRPCT was found. Thus, products for which no such studies were found were excluded from this review. These products may have evidence published in single-arm or open-label studies. However, because of the significant placebo effect of AIT and the product-specific variability of HDM AIT manufacturing,^13^^,^^14^^,^^54^ we argue that these products be regarded as experimental and hence, that their use be constrained to randomized clinical trials in which such trials are available.

Our study has several limitations. First, we excluded retrospective, single-arm, and open-label studies. The placebo effect accounts for up to 77% of the benefit in AIT trials,^54^ and both US and European regulators require that new medicinal products be subjected to DBRPCS, thus justifying our decision to include only such studies. Second, our definitions of DFS and DS were arbitrary and were based on the number of treatment arms, trial size, and trial outcomes only, without reference to other trial characteristics required by US and EU regulators. Our study categorization was designed to give a general indication of the size and purpose of each study and, therefore, the overall quality of evidence for each product. An allergist considering using any of these products should review the individual studies for each product. Third, because of the heterogeneity of study outcomes and incomplete data reporting, we were unable to perform meta-analysis. Fourth, we were able to access only study results published in the scientific literature or to the European and US Clinical Trials Registries. There are many studies registered on the European Trials Registry for which no results are available. For products that have undergone assessment by medical regulators, freedom of information requests could be used to obtain results of studies not publicly available^55^; however, for products available only on a named patient product basis, requests would need to be submitted to individual companies. Fifth, we did not review safety outcomes.

## Conclusion

We found 3 AIT products, all SLIT products, for which DFS and DS pairs had been performed to demonstrate the efficacy of the MRMD of the product in AR: the ALK 12 SQ-HDM SLIT tablet, the ALK 6 SQ-HDM tablet, and the SG 300 IR SLIT tablet. Additionally, the ALK 12 SQ-HDM SLIT tablet and ALK 6 SQ-HDM tablet have DFS and DS pairs examining their use in AA. For other products, including all subcutaneous products, the small studies identified generally had significant methodologic problems, and there was insufficient high-quality evidence to satisfy US and European medical regulators. Although the absence of such evidence does not prove that the products are ineffective, better evidence must be gathered, given the high prevalence of dust mite allergy.

## Disclosure statement

Medical writing assistance was funded by Seqirus (Australia) Pty Ltd (CSL Seqirus), the Australian distributors of ALK 6/12 SQ-HDM SLIT tablets. CSL Seqirus had no role in the systematic review, analysis, or data interpretation.

Disclosure of potential conflict of interest: C. H. Katelaris has received honoraria for presentations and fees for advisory board participation from Seqirus, as well as fees for advisory board participation from Stallegenes. T. West has received honoraria for presentations for Seqirus.

## References

[1] Savouré M., Bousquet J., Jaakkola J.J.K., Jaakkola M.S., Jacquemin B., Nadif R. (2022). Worldwide prevalence of rhinitis in adults: a review of definitions and temporal evolution. Clin Transl Allergy.

[2] Song P., Adeloye D., Salim H., Dos Santos J.P., Campbell H., Sheikh A. (2022). Global, regional, and national prevalence of asthma in 2019: a systematic analysis and modelling study. J Glob Health.

[3] Pakkasela J., Ilmarinen P., Honkamäki J., Tuomisto L.E., Andersén H., Piirilä P. (2020). Age-specific incidence of allergic and non-allergic asthma. BMC Pulm Med.

[4] Fitzhugh D.J., Lockey R.F. (2011). Allergen immunotherapy: a history of the first 100 years. Curr Opin Allergy Clin Immunol.

[5] Thomas W.R. (2010). Geography of house dust mite allergens. Asian Pac J Allergy Immunol.

[6] Calderon M.A., Casale T.B., Nelson H.S., Demoly P. (2013). An evidence-based analysis of house dust mite allergen immunotherapy: a call for more rigorous clinical studies. J Allergy Clin Immunol.

[7] Larenas-Linnemann D.E. (2012). One hundred years of immunotherapy: review of the first landmark studies. Allergy Asthma Proc.

[8] Mahler V., Esch R.E., Kleine-Tebbe J., Lavery W.J., Plunkett G., Vieths S. (2019). Understanding differences in allergen immunotherapy products and practices in North America and Europe. J Allergy Clin Immunol.

[9] Committee for Medicinal Products for Human Use (2008).

[10] Committee for Medicinal Products for Human Use (CHMP) (2008).

[11] Bonertz A., Tripathi A., Zimmer J., Reeb C., Kaul S., Bridgewater J. (2022). A regulator's view on AIT clinical trials in the United States and Europe: why successful studies fail to support licensure. J Allergy Clin Immunol.

[12] Horn A., Bachert C., Brehmer D. (2021). Clinical post-approval studies as part of the Therapy Allergen Regulation (TAV): a systematic review. Z Evid Fortbild Qual Gesundhwes.

[13] Carnés J., Iraola V., Cho S.H., Esch R.E. (2017). Mite allergen extracts and clinical practice. Ann Allergy Asthma Immunol.

[14] González-Pérez R., Poza-Guedes P., Barrios del Pino Y., Matheu V., Sánchez-Machín I. (2019). Evaluation of major mite allergens from European standardized commercial extracts for in vivo diagnosis: addressing the need for precision medicine. Clin Transl Allergy.

[15] Larenas-Linnemann D., Cox L.S. (2008). European allergen extract units and potency: review of available information. Ann Allergy Asthma Immunol.

[16] Dhami S., Nurmatov U., Arasi S., Khan T., Asaria M., Zaman H. (2017). Allergen immunotherapy for allergic rhinoconjunctivitis: a systematic review and meta-analysis. Allergy.

[17] Dhami S., Kakourou A., Asamoah F., Agache I., Lau S., Jutel M. (2017). Allergen immunotherapy for allergic asthma: a systematic review and meta-analysis. Allergy.

[18] Bush R.K., Swenson C., Fahlberg B., Evans M.D., Esch R., Morris M., Busse W.W. (2011). House dust mite sublingual immunotherapy: results of a US trial. J Allergy Clin Immunol.

[19] Demoly P., Emminger W., Rehm D., Backer V., Tommerup L., Kleine-Tebbe J. (2016). Effective treatment of house dust mite-induced allergic rhinitis with 2 doses of the SQ HDM SLIT-tablet: results from a randomized, double-blind, placebo-controlled phase III trial. J Allergy Clin Immunol.

[20] Masuyama K., Okamoto Y., Okamiya K., Azuma R., Fujinami T., Riis B. (2018). Efficacy and safety of SQ house dust mite sublingual immunotherapy-tablet in Japanese children. Allergy.

[21] Nolte H., Maloney J., Nelson H.S., Bernstein D.I., Lu S., Li Z. (2015). Onset and dose-related efficacy of house dust mite sublingual immunotherapy tablets in an environmental exposure chamber. J Allergy Clin Immunol.

[22] Nolte H., Bernstein D.I., Nelson H.S., Kleine-Tebbe J., Sussman G.L., Seitzberg D. (2016). Efficacy of house dust mite sublingual immunotherapy tablet in North American adolescents and adults in a randomized, placebo-controlled trial. J Allergy Clin Immunol.

[23] Okubo K., Masuyama K., Imai T., Okamiya K., Stage B.S., Seitzberg D. (2017). Efficacy and safety of the SQ house dust mite sublingual immunotherapy tablet in Japanese adults and adolescents with house dust mite-induced allergic rhinitis. J Allergy Clin Immunol.

[24] Virchow J.C., Backer V., Kuna P., Prieto L., Nolte H., Villesen H.H. (2016). Efficacy of a house dust mite sublingual allergen immunotherapy tablet in adults with allergic asthma: a randomized clinical trial. JAMA.

[25] Tanaka A., Tohda Y., Okamiya K., Azuma R., Terada I., Adachi M. (2020). Efficacy and safety of HDM SLIT rablet in Japanese adults with allergic asthma. J Allergy Clin Immunol Pract.

[26] Bozek A., Galuszka B., Gawlik R., Misiolek M., Scierski W., Grzanka A. (2022). Allergen immunotherapy against house dust mites in patients with local allergic rhinitis and asthma. J Asthma.

[27] Mosbech H., Deckelmann R., de Blay F., Pastorello E.A., Trebas-Pietras E., Andres L.P. (2014). Standardized quality (SQ) house dust mite sublingual immunotherapy tablet (ALK) reduces inhaled corticosteroid use while maintaining asthma control: a randomized, double-blind, placebo-controlled trial. J Allergy Clin Immunol.

[28] Mosbech H., Canonica G.W., Backer V., de Blay F., Klimek L., Broge L. (2015). SQ house dust mite sublingually administered immunotherapy tablet (ALK) improves allergic rhinitis in patients with house dust mite allergic asthma and rhinitis symptoms. Ann Allergy Asthma Immunol.

[29] Bergmann K.C., Demoly P., Worm M., Fokkens W.J., Carrillo T., Tabar A.I. (2014). Efficacy and safety of sublingual tablets of house dust mite allergen extracts in adults with allergic rhinitis. J Allergy Clin Immunol.

[30] Okamoto Y., Fujieda S., Okano M., Yoshida Y., Kakudo S., Masuyama K. (2017). House dust mite sublingual tablet is effective and safe in patients with allergic rhinitis. Allergy.

[31] Okamoto Y., Fujieda S., Okano M., Hida H., Kakudo S., Masuyama K. (2019). Efficacy of house dust mite sublingual tablet in the treatment of allergic rhinoconjunctivitis: a randomized trial in a pediatric population. Pediatr Allergy Immunol.

[32] Roux M., Devillier P., Yang W.H., Montagut A., Abiteboul K., Viatte A. (2016). Efficacy and safety of sublingual tablets of house dust mite allergen extracts: results of a dose-ranging study in an environmental exposure chamber. J Allergy Clin Immunol.

[33] Demoly P., Corren J., Creticos P., De Blay F., Gevaert P., Hellings P. (2021). A 300 IR sublingual tablet is an effective, safe treatment for house dust mite-induced allergic rhinitis: an international, double-blind, placebo-controlled, randomized phase III clinical trial. J Allergy Clin Immunol.

[34] Pham-Thi N., Scheinmann P., Fadel R., Combebias A., Andre C. (2007). Assessment of sublingual immunotherapy efficacy in children with house dust mite-induced allergic asthma optimally controlled by pharmacologic treatment and mite-avoidance measures. Pediatr Allergy Immunol.

[35] Tonnel A.B., Scherpereel A., Douay B., Mellin B., Leprince D., Goldstein N. (2004). Allergic rhinitis due to house dust mites: evaluation of the efficacy of specific sublingual immunotherapy. Allergy.

[36] SA S. (2013). https://www.clinicaltrialsregister.eu/ctr-search/search?query=2013-000487-28.

[37] Wang L., Yin J., Fadel R., Montagut A., de Beaumont O., Devillier P. (2014). House dust mite sublingual immunotherapy is safe and appears to be effective in moderate, persistent asthma. Allergy.

[38] Dokic D., Schnitker J., Narkus A., Cromwell O., Frank E. (2005). Clinical effects of specific immunotherapy: a two-year double-blind, placebo-controlled study with a one year follow-up. Prilozi.

[39] Moreno V., Alvarino M., Rodriguez F., Roger A., Pena-Arellano M.I., Lleonart R. (2016). Randomized dose-response study of subcutaneous immunotherapy with a Dermatophagoides pteronyssinus extract in patients with respiratory allergy. Immunotherapy.

[40] Bożek A., Kołodziejczyk K., Kozłowska R., Canonica G.W. (2017). Evidence of the efficacy and safety of house dust mite subcutaneous immunotherapy in elderly allergic rhinitis patients: a randomized, double-blind placebo-controlled trial. Clin Transl Allergy.

[41] Jutel M., Rudert M., Kreimendahl F., Kuna P. (2018). Efficacy and tolerability of a house dust mite allergoid in allergic bronchial asthma: a randomized dose-ranging trial. Immunotherapy.

[42] Blumberga G., Groes L., Haugaard L., Dahl R. (2006). Steroid-sparing effect of subcutaneous SQ-standardised specific immunotherapy in moderate and severe house dust mite allergic asthmatics. Allergy.

[43] Nieto A., Mazón Á., Nieto M., Ibáñez E., Jang D.T., Calaforra S. (2022). First-in-human phase 2 trial with mite allergoids coupled to mannan in subcutaneous and sublingual immunotherapy. Allergy.

[44] A multicentre randomised placebo-controlled double-blind clinical trial for evaluation of safety and efficacy of a specific immunotherapy with an aluminium hydroxide-adsorbed allergoid preparation. EU Clinical Trials Register. https://www.clinicaltrialsregister.eu/ctr-search/search?query=2006-000934-11.

[45] Pfaar O., Nell M.J., Boot J.D., Versteeg S.A., van Ree R., Roger A. (2016). A randomized, 5-arm dose finding study with a mite allergoid SCIT in allergic rhinoconjunctivitis patients. Allergy.

[46] A multicentre randomised placebo-controlled double-blind clinical trial for evaluation of safety and efficacy of specific immunotherapy with an aluminium hydroxide-adsorbed allergoid preparation of birch pollen allergens. Allergopharma Gmmh and Company. KG. (EudraCT 2004-003892-35): EU Clinical Trials Register. https://www.clinicaltrialsregister.eu/ctr-search/search?query=2004-003892-35.

[47] Roberts G., Pfaar O., Akdis C.A., Ansotegui I.J., Durham S.R., Gerth van Wijk R. (2018). EAACI guidelines on allergen immunotherapy: allergic rhinoconjunctivitis. Allergy.

[48] Passalacqua G., Albano M., Fregonese L., Riccio A., Pronzato C., Mela G.S. (1998). Randomised controlled trial of local allergoid immunotherapy on allergic inflammation in mite-induced rhinoconjunctivitis. Lancet.

[49] Ameal A., Vega-Chicote J.M., Fernández S., Miranda A., Carmona M.J., Rondón M.C. (2005). Double-blind and placebo-controlled study to assess efficacy and safety of a modified allergen extract of Dermatophagoides pteronyssinus in allergic asthma. Allergy.

[50] KG A.G.C. A multicenter randomized double-blind placebo-controlled clinical trial for evaluation of efficacy and safety of specific immunotherapy with an aluminium hydroxide-adsorbed allergoid preparation of house dust mite (Dermatophagoides pteronyssinus) in patients with allergic bronchial asthma and with allergic rhinitis or rhinoconjunctivitis. (EudraCT 2015-000188-15): EU Clinical Trials Register. https://www.clinicaltrialsregister.eu/ctr-search/trial/2015-000188-15/results.

[51] B.V. HA (2021). https://www.clinicaltrialsregister.eu/ctr-search/trial/2016-000051-27/results.

[52] Hüser C., Dieterich P., Singh J., Shah-Hosseini K., Allekotte S., Lehmacher W. (2017). A 12-week DBPC dose-finding study with sublingual monomeric allergoid tablets in house dust mite-allergic patients. Allergy.

[53] Passalacqua G., Pasquali M., Ariano R., Lombardi C., Giardini A., Baiardini I. (2006). Randomized double-blind controlled study with sublingual carbamylated allergoid immunotherapy in mild rhinitis due to mites. Allergy.

[54] Pfaar O., Gerth van Wijk R., Klimek L., Bousquet J., Creticos P.S. (2020). Clinical trials in allergen immunotherapy in the age group of children and adolescents: current concepts and future needs. Clin Transl Allergy.

[55] Kirsch I. (2014). Antidepressants and the placebo effect. Z Psychol.

[56] Mauro M., Boni E., Makri E., Incorvaia C. (2015). Pharmacodynamic and pharmacokinetic evaluation of house dust mite sublingually administered immunotherapy tablet in the treatment of asthma. Expert Opin Drug Metab Toxicol.

[57] Bernstein D.I., Kleine-Tebbe J., Nelson H.S., Bardelas J.A., Sussman G.L., Lu S. (2018). SQ house dust mite sublingual immunotherapy tablet subgroup efficacy and local application site reaction duration. Ann Allergy Asthma Immunol.

[58] Demoly P., Okamoto Y., Yang W.H., Devillier P., Bergmann K.C. (2016). 300 IR HDM tablet: a sublingual immunotherapy tablet for the treatment of house dust mite-associated allergic rhinitis. Expert Rev Clin Immunol.

[59] Aydogan M., Eifan A.O., Keles S., Akkoc T., Nursoy M.A., Bahceciler N.N. (2013). Sublingual immunotherapy in children with allergic rhinoconjunctivitis mono-sensitized to house-dust-mites: a double-blind-placebo-controlled randomised trial. Respir Med.

[60] Standardized mite Dermatophagoides farinae - Dermatophagoides farinae concentrate Standardized mite mix Dermatophagoides farinae and Dermatophagoides pteronyssinus - Dermatophagoides farinae and Dermatophagoides pteronyssinus concentrate. Standardized mite Dermatophagoides pteronyssinus- Dermatophagoides pteronyssinus concentrate. Greer Laboratories, Inc. https://dailymed.nlm.nih.gov/dailymed/drugInfo.cfm?setid=9b7f878e-613f-4831-9223-a6a07fb09f1b&audience=consumer.

[61] Di Gioacchino M., Cavallucci E., Ballone E., Cervone M., Di Rocco P., Piunti E. (2012). Dose-dependent clinical and immunological efficacy of sublingual immunotherapy with mite monomeric allergoid. Int J Immunopathol Pharmacol.

[62] Klimek L., Fox G.-C., Thum-Oltmer S. (2018). SCIT with a high-dose house dust mite allergoid is well tolerated: safety data from pooled clinical trials and more than 10 years of daily practice analyzed in different subgroups. Allergo J Int.

[63] Zimmer J., Schmidt S., Kaul S., Costanzo A., Buchheit K.-H., Brown S. (2022). Standardisation of allergen products: 4. Validation of a candidate European Pharmacopoeia standard method for quantification of major grass pollen allergen Phl p 5. Allergy.

[64] Bahçeciler N.N., Işik U., Barlan I.B., Başaran M.M. (2001). Efficacy of sublingual immunotherapy in children with asthma and rhinitis: a double-blind, placebo-controlled study. Pediatr Pulmonol.

[65] Bozek A., Ignasiak B., Filipowska B., Jarzab J. (2013). House dust mite sublingual immunotherapy: a double-blind, placebo-controlled study in elderly patients with allergic rhinitis. Clin Exp Allergy.

[66] Guez S., Vatrinet C., Fadel R., André C. (2000). House-dust-mite sublingual-swallow immunotherapy (SLIT) in perennial rhinitis: a double-blind, placebo-controlled study. Allergy.

[67] Mortemousque B., Bertel F., De Casamayor J., Verin P., Colin J. (2003). House-dust mite sublingual-swallow immunotherapy in perennial conjunctivitis: a double-blind, placebo-controlled study. Clin Exp Allergy.

[68] O'Hehir R.E., Gardner L.M., de Leon M.P., Hales B.J., Biondo M., Douglass J.A. (2009). House dust mite sublingual immunotherapy: the role for transforming growth factor-beta and functional regulatory T cells. Am J Respir Crit Care Med.

[69] Potter P.C., Baker S., Fenemore B., Nurse B. (2015). Clinical and cytokine responses to house dust mite sublingual immunotherapy. Ann Allergy Asthma Immunol.

[70] Tseng S.H., Fu L.S., Nong B.R., Weng J.D., Shyur S.D. (2008). Changes in serum specific IgG4 and IgG4/ IgE ratio in mite-sensitized Taiwanese children with allergic rhinitis receiving short-term sublingual-swallow immunotherapy: a multicenter, randomized, placebo-controlled trial. Asian Pac J Allergy Immunol.

[71] Niu C.K., Chen W.Y., Huang J.L., Lue K.H., Wang J.Y. (2006). Efficacy of sublingual immunotherapy with high-dose mite extracts in asthma: a multi-center, double-blind, randomized, and placebo-controlled study in Taiwan. Respir Med.

[72] Bousquet J., Scheinmann P., Guinnepain M.T., Perrin-Fayolle M., Sauvaget J., Tonnel A.B. (1999). Sublingual-swallow immunotherapy (SLIT) in patients with asthma due to house-dust mites: a double-blind, placebo-controlled study. Allergy.

[73] Lue K.H., Lin Y.H., Sun H.L., Lu K.H., Hsieh J.C., Chou M.C. (2006). Clinical and immunologic effects of sublingual immunotherapy in asthmatic children sensitized to mites: a double-blind, randomized, placebo-controlled study. Pediatr Allergy Immunol.

[74] Chen Y., Zhou L., Yang Y. (2017). Effect of sublingual immunotherapy on platelet activity in children with allergic rhinitis. Braz J Otorhinolaryngol.

[75] Luo R., Liu W., Wang J., Chen Y., Sun C., Zhou L. (2014). Role of BAFF in pediatric patients with allergic rhinitis during sublingual immunotherapy. Eur J Pediatr.

[76] Wang C., Wang K., Liu S., Qin X., Chen K., Zhang T. (2016). Decreased level of osteopontin in children with allergic rhinitis during sublingual immunotherapy. Int J Pediatr Otorhinolaryngol.

[77] Tian M., Wang Y., Lu Y., Jiang Y.H., Zhao D.Y. (2014). Effects of sublingual immunotherapy for Dermatophagoides farinae on Th17 cells and CD4(+) CD25(+) regulatory T cells in peripheral blood of children with allergic asthma. Int Forum Allergy Rhinol.

[78] KG A.J.G. (2006). https://www.clinicaltrialsregister.eu/ctr-search/search?query=eudract_number:2006-000934-11.

[79] Varney V.A., Tabbah K., Mavroleon G., Frew A.J. (2003). Usefulness of specific immunotherapy in patients with severe perennial allergic rhinitis induced by house dust mite: a double-blind, randomized, placebo-controlled trial. Clin Exp Allergy.

[80] Xian M., Feng M., Dong Y., Wei N., Su Q., Li J. (2020). Changes in CD4+CD25+FoxP3+ regulatory T cells and serum cytokines in sublingual and subcutaneous immunotherapy in allergic rhinitis with or without asthma. Int Arch Allergy Immunol.

[81] Wang H., Lin X., Hao C., Zhang C., Sun B., Zheng J. (2006). A double-blind, placebo-controlled study of house dust mite immunotherapy in Chinese asthmatic patients. Allergy.

[82] García-Robaina J.C., Sánchez I., de la Torre F., Fernández-Caldas E., Casanovas M. (2006). Successful management of mite-allergic asthma with modified extracts of Dermatophagoides pteronyssinus and Dermatophagoides farinae in a double-blind, placebo-controlled study. J Allergy Clin Immunol.

[83] (2013). Safety and efficacy of house dust mite allergen extract in the treatment of allergic rhinoconjunctivitis (NCT00263549).

[84] Yukselen A., Kendirli S.G., Yilmaz M., Altintas D.U., Karakoc G.B. (2012). Effect of one-year subcutaneous and sublingual immunotherapy on clinical and laboratory parameters in children with rhinitis and asthma: a randomized, placebo-controlled, double-blind, double-dummy study. Int Arch Allergy Immunol.

[85] Zieglmayer P., Nolte H., Nelson H.S., Bernstein D.I., Kaur A., Jacobi H. (2016). Long-term effects of a house dust mite sublingual immunotherapy tablet in an environmental exposure chamber trial. Ann Allergy Asthma Immunol.

[86] Wen Y., Zhou L., Li Y., Li Z., Deng W., Zhang T. (2018). Role of leptin in allergic rhinitis during sublingual immunotherapy. Eur Arch Otorhinolaryngol.

[87] (2013). Multicenter trial of immunotherapy with house dust mite allergoid (ACRI) (NCT00540631).

[88] (2015). Safety and efficacy of house dust mite allergoid in the treatment of bronchial asthma (NCT00263640).

[89] Zielen S., Kardos P., Madonini E. (2010). Steroid-sparing effects with allergen-specific immunotherapy in children with asthma: a randomized controlled trial. J Allergy Clin Immunol.

