# Supplementary material for: The evidence for commercial house dust mite immunotherapy products: A pragmatic systematic review with narrative synthesis
Source: J Allergy Clin Immunol Glob. 2024 Apr 10;3(3):100255. doi: 10.1016/j.jacig.2024.100255 (PMC11292502; doi:10.1016/j.jacig.2024.100255)
Supplement: Supplementary Data [file mmc1.docx]

**Appendices: The evidence for commercial house dust mite immunotherapy products: a pragmatic systematic review with narrative synthesis**

**Search strategy**

| **Search strategy - Medline and Embase** | | |
| --- | --- | --- |
| 1 | RCTs | "randomized controlled trial".pt. or "controlled clinical trial.pt".mp. or "randomized".ab. or "placebo".ab. or "drug therapy".fs. or "randomly".ab. or "trial".ab. or "groups".ab. [mp=title, abstract, original title, name of substance word, subject heading word, floating sub-heading word, keyword heading word, organism supplementary concept word, protocol supplementary concept word, rare disease supplementary concept word, unique identifier, synonyms] |
| 2 | HDM | (Dermatophagoides or pteronyssinus or farinae or mite).ti,ab. |
| 3 | Diseases | ("asthma" or "allerg*").ti,ab. |
| 4 | AIT | (Immunotherap* or sublingual* or subcutaneous* or allergoid or vaccin* or "allergen extract" or SLIT or AIT or SIT or SCIT).ti,ab |
| 5 |  | 1 and 2 and 3 and 4 |
|  |  | limit 5 to (english language and humans and yr="2000-Current") |
|  |  |  |
| **Search Strategy - ClinicalTrials.gov** | | |
|  | Condition | allergic rhinitis OR allergic rhinoconjunctivitis OR allergic conjunctivitis OR asthma OR House Dust Mite Allergy OR Allergic Sensitisation OR Allergic Condition OR Allergy In Children OR Allergy |
|  | Other terms | Dermatophagoides OR pteronyssinus OR farinae OR mite |
|  | Phase | 1, 2 or 3 |
|  |  |  |
| **Search Strategy - EU Clinical Trials register (https://www.clinicaltrialsregister.eu)** | | |
|  |  | (allergic rhinitis OR allergic rhinoconjunctivitis OR allergic conjunctivitis OR asthma) AND (Dermatophagoides OR pteronyssinus OR farinae OR mite) |
|  | Phase | 1, 2 or 3 |
|  |  | THE ABOVE DOES NOT FIND ALL STUDIES - instead JUST search for: Dermatophagoides OR pteronyssinus OR farinae OR mite |
|  |  |  |

**Extracted data items**

Extracted data included:

1. The country or countries in which the study was performed.
2. The mean and/or median age and variance (SD and/or range) in each treatment arm (active and placebo).
3. The allergen sensitization test methods(s) used as inclusion criteria: skin prick testing (SPT); serum specific IgE assay; nasal or bronchial provocation tests, etc.
4. The presence of absence of allergic rhinitis (AR) and allergic asthma (AA) in the patient population. Trials of AR will be analyzed separately from those of AA.
5. The criteria for disease severity on inclusion.
6. The manufacturer and brand name of the AIT formulation and its overall formulation: mite species; aqueous vs alum; native allergen vs allergoid; presence of other components); concentration of allergen (measured in ug/mL of major allergen and/or proprietary units of the manufacturer).
7. The administration regimen and initiation schedule/up-dosing phase if any.
8. The maintenance dose and calculated cumulative dose administered (measured in ug/mL of major allergen and/or proprietary units of the manufacturer).
9. The mean treatment time (in months).
10. The duration of any baseline period prior to initiation of AIT, and of any monitoring period following completion of the treatment period.
11. The total number of randomized patients, intention-to-treat and per-protocol populations in the active and placebo arms.
12. The primary outcome definition, including the minimal clinically relevant difference in the outcome, and the justification for this decision.
13. The primary and secondary efficacy criteria and results for each treatment arm, including both clinical assessment scales and quality of life scales.
14. The nature and number of symptoms scored, symptom scale and variance for active and placebo groups, and the reported p value for active vs placebo groups.
15. The combined symptom and medication score, overall symptom score for active and placebo groups, and the p value for active vs placebo groups.

**Table A1. Information on published papers and registered studies obtained from AIT manufacturers and other sources.**

| **Study** | **Source of information** | **Information obtained from manufacturer** |
| --- | --- | --- |
| Ameal et al. 2005 (1) | LETI Pharma, S.L.U.; Spain | The IMP in this study is Depigoid, which is commercially available in several European countries. The maintenance dose in the study, 0.5 mL of vial 2 (14.25 ug of Der p1/ml and 8.61 of Der p2/mL), corresponds to the current maintenance dose of the 100% DP formulation of Depigoid, as per the PI (0.5 mL of 100 DPP/mL formulation). |
| Andri et al. 1993 (2) | LOFARMA S.p.A., Italy | The IMP in this study, Allerkin Intranasal, is no longer commercially available. |
| Aydogan et al. 2013 (3) | Stallergenes Greer International AG; Switzerland | The IMP in this study is Staloral (300 IR/mL SLIT liquid). The study used a 100 uL dropper. Patients in this study received a maintenance dose of 240 IR per day (8 'drops' at 100 μL per 'drop' of a 300 IR/mL solution), taken 3 days per week. |
| BJDC 1979 (4) | Bencard Allergie GmbH, Germany | Study is contemporaneous with Bencard Migen papers. Bencard confirmed Migen is not available as a commercial product. This study was assumed to refer either to Migen, to another product which no longer exists, or to unbranded extracts. The manufacturer did not reply to further enquiries to confirm this. |
| Blainey et al. 1984 (5) | Bencard Allergie GmbH, Germany | The IMP in this study, Bencard Migen, is no longer commercially available. |
| Bousquet et al. 1999 (6) | Stallergenes Greer International AG; Switzerland | The IMP in this study is Staloral (300 IR/mL SLIT liquid). This study used a 50 μL dropper. Patients in this study received a maintenance dose of 300 IR per day (20 'drops', at 50 μL per 'drop' of a 300 IR/mL solution), taken 3 days per week. |
| Bozek et al. 2013 (7) | Stallergenes Greer International AG; Switzerland | The IMP in this study is Staloral (300 IR/mL SLIT liquid). As stated in the paper, the dose of IMP administered in the trial was 540 IR (18 ‘puffs’ at 30 IR per ‘puff’), 5 days per week. The dose information in the US Clinical Trials Database (NCT01605760) is not correct. |
| Bozek et al. 2017 (8) | HAL Allergy B.V., The Netherlands | The IMP in this study was the DP 50% and DF 50% formulation of Purethal Mites SCIT. This formulation is currently commercially available. |
| Bozek et al. 2022 (9) | ALK-Abelló A/S, Denmark | The IMP in this study was the ALK-Abelló 12 SQ-HDM tablet |
| Buchanan et al. 1980 (10) | Bencard Allergie GmbH, Germany | The IMP in this study, Bencard Migen, is no longer commercially available. |
| Bush et al. 2011 (11) | Stallergenes Greer International AG; Switzerland | The IMP in this study is bulk Stallergenes-Greer glycerinated HDM extracts. In the United States, these extracts are standardized by the FDA for clinical use. |
| Chen et al. 2017 (12) | Zhejiang Wolwo Bio-Pharmaceutical Co Ltd, China | The IMP in this study is Chanllergen. In the paper, it is referred to by its generic name, "Dermatophagoides Farinae Drops". |
| de Bot et al. 2012 (13) | ALK-Abelló A/S, Denmark | The IMP in this study was Oralgen Mijten. This product is no longer commercially available. |
| Dokic et al. 2005 (14) | Allergopharma GmbH & Co. KG; Germany | The IMP in this study was Acaroid, which is commercially available in several European countries. This study had internal company study ID 97-09 M. Most patients in this study were given the IMP every four weeks. A mean of 30 doses of IMP were given in both study groups. The dose in the paper is written in units of PNU/mL whereas the product information for Acaroid uses units of TU/mL. PNU/mL can be converted to TU/mL by multiplying by 3.333. The maintenance dose in the study of 0.6 mL of 3000 PNU/mL solution corresponds to 6000 TU per dose, which is the current MRMD. |
| D'Souza et al. 1973 (15) | Bencard Allergie GmbH, Germany | The IMP in this study were unbranded allergen extracts manufactured by Beecham Research Laboratories, now part of Bencard. The IMP in this study is not a commercially available product. |
| Franco et al. 1995 (16) | Stallergenes Greer International AG; Switzerland | The IMP in this study, Alpare Mite, is no longer commercially available. |
| Gabriel et al. 1977 (17) | Bencard Allergie GmbH, Germany | The IMP in this study, Bencard Migen, is no longer commercially available. |
| Gaddie et al. 1976 (18) | Bencard Allergie GmbH, Germany | The IMP in this study, Bencard Migen, is no longer commercially available. |
| Garcia-Robaina et al. 2006 (19) | LETI Pharma, S.L.U.; Spain | The IMP in this study is Depigoid, which is commercially available in several European countries. The maintenance dose in the study, 0.5 mL of vial 2 (35 mg/mL of DP plus 28 mg of DF), corresponds to the current maintenance dose of the 50% DP, 50% DF formulation of Depigoid, as per the PI (0.5 mL of 100 DPP/mL formulation). |
| Giovane et al. 1994 (20) | Allergopharma GmbH & Co. KG; Germany | The IMP in this study was Novohelisen Oral. This product is no longer commercially available. |
| Grembiale et al. 2000 (21) | Calderon 2013 (cite) | The IMP in this study, Conjuvac SCIT, is no longer commercially available. |
| Guez et al. 2000 (22) | Stallergenes Greer International AG; Switzerland | The IMP in this study is Staloral (300 IR/mL SLIT liquid). This study used a 50 μL dropper. Patients in this study received a maintenance dose of 300 IR per day (20 'drops', at 50 μL per 'drop' of a 300 IR/mL solution), taken 3 days per week. |
| Guo et al. 2017 (23) | ALK-Abelló A/S, Denmark | The IMP in this study was Pangramin Plus SCIT, also known as Pangramin Depot SCIT. This product is no longer commercially available. |
| HAL Allergy B.V. (EudraCT 2016-000051-27) (24) | HAL Allergy B.V., The Netherlands | The IMP in this study was the DP 50% and DF 50% formulation of Purethal Mites SCIT. This formulation is currently commercially available. |
| Hoseini et al. 2018 (25) | Zhejiang Wolwo Bio-Pharmaceutical Co Ltd, China | The IMP in this study is Chanllergen. In the paper, it is referred to by its generic name, "Dermatophagoides Farinae Drops". |
| Ippoliti et al. 2003 (26) | ALK-Abelló A/S, Denmark | The IMP in this study was Pangramin Plus SCIT, also known as Pangramin Depot SCIT. This product is no longer commercially available. |
| Jutel et al. 2018 (27) | Allergopharma GmbH & Co. KG; Germany | The paper Jutel 2018 corresponded to internal Allergopharma study ID Al1009ac. The study was registered in the European Clinical Trials Database with EudraCT number 2011-002248-29. The IMP was administered every four weeks in most circumstances. The median number of doses received per patient was 15 of 16 planned doses. |
| Lozano et al. 2014 (28) | HAL Allergy B.V., The Netherlands | The IMP in this study was the DP 50% and DF 50% formulation of Purethal Mites SCIT. This formulation is currently commercially available. |
| Lue et al. 2006 (29) | Stallergenes Greer International AG; Switzerland | The IMP in this study is Staloral (300 IR/mL SLIT liquid). Patients in this study received a maintenance dose of 300 IR per day (20 ‘drops’, at 50 μL per ‘drop’, of a 300 IR/mL solution), taken 7 days per week. |
| Maestrelli et al. 2004 (30) | ALK-Abelló A/S, Denmark | The IMP in this study was Pangramin Plus SCIT, also known as Pangramin Depot SCIT. This product is no longer commercially available. |
| Marcucci et al. 2005 (31) | ALK-Abelló A/S, Denmark | The IMP in this study was SlitONE. This product is no longer commercially available. |
| Maunsell et al. 1971 (32) | Bencard Allergie GmbH, Germany | Study is contemporaneous with Bencard Migen papers. Bencard confirmed Migen is not available as a commercial product. This study was assumed to refer either to Migen, to another product which no longer exists, or to unbranded extracts. The manufacturer did not reply to further enquiries to confirm this. |
| Moreno et al. 2016 (33) | Roxall Medizin GmbH; Germany | The IMP in this study is Allergovac Depot, which is commercially available in several European countries. Group 2 of the study (maintenance dose 0.5 mL of 0.125 SPT formulation) corresponds to the current commercial dose (1000 TSU, i.e., 1 mL of 1,000 TSU/mL formulation). The extracts in the study were concentrated such that 0.5 mL of IMP in the study yielded a dose of 1000 TSU). |
| Mortemousque et al. 2003 (34) | Stallergenes Greer International AG; Switzerland | The IMP in this study is Staloral (300 IR/mL SLIT liquid). The study used a 50 uL dropper. Patients in this study received a maintenance dose of 300 IR per day (20 ‘drops’, at 50 μL per ‘drop’ of a 300 IR/mL solution), taken 3 days per week. |
| Mungan et al. 1999 (35) | Stallergenes Greer International AG; Switzerland | The IMP in this study is Stallergenes-Greer Staloral. The study was single-blinded only, not double-blinded. The dose was 100 IR/day, lower than the current MRMD of 300 IR/day. |
| Nieto et al. 2022 (36) | Inmunotek SL; Spain | The SLIT product used in this study is commercially available in several countries and is branded SLIMtdc. This is a mannan-bound allergoid product, which is a different product to other Inmunotek allergoids such as Acaroid, Clustek and Clustek Max, which are all alum-adsorbed products. |
| Niu et al. 2006 (37) | Stallergenes Greer International AG; Switzerland | The IMP in this study is Staloral (300 IR/mL SLIT liquid). The study used a 50 uL dropper. Patients in this study received a maintenance dose of 300 IR per day (20 ‘drops’, at 50 μL per ‘drop’, of a 300 IR/mL solution), taken 7 days per week. |
| O'Hehir et al. 2009 (38) | Stallergenes Greer International AG; Switzerland | The IMP in this study is Staloral (300 IR/mL SLIT liquid). The study used a 100 uL dropper. Patients in this study received a maintenance dose of 240 IR per day (8 'drops' at 100 μL per 'drop' of a 300 IR/mL solution), taken 7 days per week. |
| Pajno et al. 2000 (39) | ALK-Abelló A/S, Denmark | The IMP in this study was SlitONE. This product is no longer commercially available. |
| Pauli et al. 1984 (40) | Bencard Allergie GmbH, Germany | The IMP in this study, Bencard Migen, is no longer commercially available. |
| Peroni et al. 1995 (41) | Stallergenes Greer International AG; Switzerland | The IMP in this study, Alpare Mite, is no longer commercially available. |
| Queiros et al. 2013 (42) | FDA Allergenic Ldta, Brazil | No response received from study author or manufacturer. The IMP in the study was presumed to not be a commercially available product. |
| Riechelmann et al. 2010 (43) | HAL Allergy B.V., The Netherlands | The IMP in this study was the 100% DP formulation of Purethal Mites. This formulation is no longer commercially available; Purethal Mites is now manufactured as 50% DP, 50% DF. |
| Rodriguez et al. 2006 (44) | ALK-Abelló A/S, Denmark | The IMP in this study was SlitONE. This product is no longer commercially available. |
| Rondon et al. 2016 (45) | ALK-Abelló A/S, Denmark | The IMP in this study was Pangramin Plus SCIT, also known as Pangramin Depot SCIT. This product is no longer commercially available. |
| Smith et al. 1972 (46) | Bencard Allergie GmbH, Germany | The IMP in this study was unbranded allergen extracts manufactured by Beecham Research Laboratories, now part of Bencard. The IMP in this study is not a commercially available product. |
| Tari et al. 1990 (47) | ALK-Abelló A/S, Denmark | The IMP in this study was Pangramin Plus SLIT. This product is no longer commercially available with HDM allergens. |
| Tian et al. 2014 (48) | Zhejiang Wolwo Bio-Pharmaceutical Co Ltd, China | The IMP in this study is Chanllergen. In the paper, it is referred to by its generic name, "Dermatophagoides Farinae Drops". |
| Tseng et al. 2008 (49) | Stallergenes Greer International AG; Switzerland | The IMP in this study is Staloral (300 IR/mL SLIT liquid). The study used a 50 uL dropper. Patients in this study received a maintenance dose of 300 IR per day (20 'drops' at 50 μL per 'drop' of a 300 IR/mL solution). The frequency of dosing is not stated in the paper, but it can be inferred to be less frequently than daily, because the study states that the cumulative dose, if taken correctly was 37312 IR. However, if a dose of 300 IR/day was taken daily for 21 weeks, this would yield a cumulative dose of 44100 IR. |
| Vidal et al. 2011 (50) | ALK-Abelló A/S, Denmark | The IMP in this study was Pangramin Plus SCIT, also known as Pangramin Depot SCIT. This product is no longer commercially available. |
| Wang et al. 2013 (51) | Zhejiang Wolwo Bio-Pharmaceutical Co Ltd, China | The IMP in this study was never commercialized. It is not, and was never, commercially available. |
| Warner et al. 1978 (52) | Bencard Allergie GmbH, Germany | The IMP in this study, Bencard Migen, is no longer commercially available. |
| Yukselen et al. 2012 (53) | Allergopharma GmbH & Co. KG; Germany | The IMP in this study was Novohelisen Oral. This product is no longer commercially available. |
| Unpublished study (US trial registration: NCT01115595) | ALK-Abelló A/S, Denmark | Queries submitted (1) to confirm the product used in this study, and (2) to determine if results could be obtained, since the study is marked as 'complete' on the US clinical trials database, but no results are published there. ALK-Abelló advised that based on the dose description, this study likely uses ALK bulk commercial SCIT extracts which are sold in some markets including the United States. ALK-Abelló was not involved in the trial and does not have the results. No reply was received from the study investigators. |
| Unpublished study (EU trial registration: 2005-001573-10) | ALK-Abelló A/S, Denmark | The IMP in this study was Pangramin Plus SCIT, also known as Pangramin Depot SCIT. This product is no longer commercially available. |
| Unpublished study (EU trial registration 2005-004731-21; US trial registration: NCT00633919) | ALK-Abelló A/S, Denmark | The IMP in this study, SLITone DP/DF, is no longer commercially available. SLITone is distinct from SLIToneULTRA, which is available in several countries. |
| Unpublished study (US trial registration: NCT00263549) | Allergopharma GmbH & Co. KG; Germany | Results of this study were published as a conference poster presentation at EAACI 2006 (Abstract Book EAACI 2006 2006:227) but was not published in the peer-reviewed scientific literature. The manufacturer was able to provide the poster. |
| Unpublished study (EU trial registration 2004-003892-35; US trial registration: NCT00263640) | Allergopharma GmbH & Co. KG; Germany | In children, the study was open label, i.e., not blinded and not placebo controlled. These results have been published in the peer-reviewed scientific literature (54). In adults, the study was double blind and placebo controlled. The results are published to the European trials database but are not published elsewhere. The study was negative in adults. The internal company study ID for this study was Al0104av. The maintenance dose for the study was 6000 TU every 4-6 weeks, with most patients receiving the dose every 4 weeks. |
| Unpublished study (EU trial registration 2006-000934-11; US trial registration: NCT00540631) | Allergopharma GmbH & Co. KG; Germany | Results of this study are published to the European trials database but have not been published in the peer-reviewed scientific literature. The internal company study ID for this study was AL0106ac. |
| Unpublished study (EU trial registration: 2015-000188-15) | Allergopharma GmbH & Co. KG; Germany | Results of this study are published to the European trials database and were presented at the 2020 European Allergy and Clinical Immunology conference (55) but have not been published in the peer-reviewed scientific literature. |
| Unpublished study (US trial registration: NCT01179282) | Responsible Party as per US Clinical Trials site listing. | The study never begun. No patients were ever enrolled. |
| Unpublished study (EU trial registration: 2016-000051-27) | HAL Allergy B.V., The Netherlands | The IMP in this study was the DP 50% and DF 50% formulation of Purethal Mites SCIT. This formulation is currently commercially available. Results for this study are published to the European Clinical Trials Registry but have not been published in the peer-reviewed scientific literature. |

*IMP, investigational medical product; DP, Dermatophagoides pteronyssinus; PI, product information; DPP, depigmented polymerized unit; IR, index of reactivity; SLIT, sublingual immunotherapy; SCIT, subcutaneous immunotherapy; SQ-HDM, standardized quality-house dust mite; HDM, house dust mite; PNU, protein nitrogen units; TU, therapeutic units; DF, Dermatophagoides farinae; SPT, skin prick test; TSU, treatment standardized unit; MRMD, manufacturer-recommended maintenance dose.*

**Table A2. Select papers excluded from our review and reason for exclusion.**

| **Study** | **Reason for exclusion** |
| --- | --- |
| ALK-Abelló, S.A.  (EudraCT: 2005 004731-21; Protocol # 2005-004731-21) (56) | The manufacturer advised that the IMP used in this study, Pangramin Plus SCIT (also known as Pangramin Depot SCIT) is no longer commercially available. |
| ALK-Abelló (NCT01603056) (57) | The manufacturer advised that the IMP used in this study, Pangramin SLIT is no longer commercially available. |
| Alvarez et al. 2002 (58) | The IMP used in the study is not a commercially manufactured AIT product designed for clinical use. |
| Andri et al. 1993 (2) | The manufacturer advised that the IMP used in this study, Allerkin Intranasal, is no longer commercially available. |
| Arikan et al. 2004 (59) | No placebo arm (study is SLIT vs SLIT + Bacillus Calmette–Guérin vaccination). |
| Baris et al. 2014 (60) | No placebo arm (study is SCIT vs SCIT + vitamin D). |
| Basomba et al. 2002 (61) | The IMP used in the study is not a commercially manufactured AIT product designed for clinical use. |
| BJDC 1979 (4) | The IMP used in this study was presumed to be Bencard Migen; see Table A1. The manufacturer advised that Bencard Migen is no longer commercially available. |
| Blainey et al. 1984 (5) | The manufacturer advised that the IMP used in this study, Bencard Migen, is no longer commercially available. |
| Bousquet et al. 1985 (62) | Study duration was seven weeks, less than the threshold of 3 months for inclusion in our review. |
| Branco Ferreira et al. 2005 (63) | Not placebo controlled (open label study). |
| Buchanan et al. 1980 (10) | The manufacturer advised that the IMP used in this study, Bencard Migen, is no longer commercially available. |
| Casanovas et al. 2005 (64) | Study does not contain a placebo arm; both arms of the study were actively treated. |
| Chen et al. 2009 (65) | Not placebo controlled (open label study). |
| Chen et al. 2020 (66) | Not placebo controlled. |
| Chen et al. 2020 (67) | Not placebo controlled. |
| Chen et al. 2020 (68) | Not placebo controlled. |
| Circassia Limited 2014 (EudraCT 2014-001662-94; Protocol #: TH005) (69) | Product was never commercially marketed, and development has now ceased (70). |
| Corzo et al. 2014 (71) | Study duration was 28 days, less than the threshold of 3 months for inclusion in our review. No clinical outcomes reported. |
| Cosmi et al. 2006 (72) | Not placebo controlled. |
| de Bot et al. 2012 (13) | The manufacturer advised that the IMP used in this study, Oralgen Mijten, is no longer commercially available. |
| Didier et al. 2015 (73) | Not placebo controlled. |
| Dreborg et al. 2012 (74) | No placebo arm (study is dust mite vs Timothy grass). |
| D’Souza et al. 1973 (15) | The manufacturer advised that the IMP used in this study, unbranded allergen extracts manufactured by Beecham Research Laboratories, is no longer commercially available. |
| Eifan et al. 2010 (75) | Not placebo controlled (open label study). |
| Fan et al. 2017 (76) | No placebo group (both arms of trial were actively treated). |
| Fanales-Belasio et al. 1995 (77) | The manufacturer advised that the IMP used in this study, Allerkin Intranasal, is no longer commercially available. |
| Feng et al. 2020 (78) | Not a randomized clinical trial. |
| Feng et al. 2022 (79) | Not a randomized clinical trial. |
| Ferreira et al. 2005 (80) | No placebo group (both arms of trial were actively treated). |
| Ferrer et al. 2003 (81) | Not placebo controlled (open label study). |
| Franco et al. 1995 (16) | The manufacturer advised that the IMP used in this study, Alpare Mite, is no longer commercially available. |
| Gabriel et al. 1977 (17) | The manufacturer advised that the IMP used in this study, Bencard Migen, is no longer commercially available. |
| Gaddie et al. 1976 (18) | The manufacturer advised that the IMP used in this study, Bencard Migen, is no longer commercially available. |
| Gardner et al. 2004 (82) | Not placebo controlled (open label study). |
| Di Gioacchino et al. 2012 (83) | Not placebo controlled. |
| Giovane et al. 1994 (20) | The manufacturer advised that the IMP used in this study, Novohelisen Oral, is no longer commercially available. |
| Grembiale et al. 2000 (21) | The manufacturer advised that the IMP used in this study, Conjuvac SCIT, is no longer commercially available. |
| Guo et al. 2017 (23) | The manufacturer advised that the IMP used in this study, Pangramin Plus SCIT, is no longer commercially available. |
| Hoseini et al. 2018 (25) | Paper poorly written with internal inconsistencies. Abstract states 40 patients enrolled whereas methods section states 47 patients enrolled. |
| Hoshino et al. 2019 (84) | Not placebo controlled. |
| Hoshino et al. 2020 (85) | Not placebo controlled. |
| Hoshino et al. 2021 (86) | Not placebo controlled. |
| Hui et al. 2014 (87) | Not placebo controlled. |
| Ibero & Castillo 2006 (88) | Not placebo controlled (open label study). |
| Ippoliti et al. 2003 (26) | The manufacturer advised that the IMP used in this study was Pangramin Plus SCIT (also known as Pangramin Depot SCIT). This product is no longer commercially available. |
| Keles et al. 2011 (89) | Not placebo controlled. |
| Kim et al. 2018 (90) | Not placebo controlled. |
| Kim et al. 2021 (91) | Not placebo controlled. |
| Królewicz et al. 2016 (92) | Not placebo controlled (open label study). |
| La Grutta et al. 2007 (93) | Not placebo controlled. |
| Lauriello et al. 2005 (94) | Not placebo controlled. |
| Lewith et al. 2002 (95) | Homeopathic treatment; not a commercially available product. |
| Li et al. 2016 (96) | Not placebo controlled. |
| Li et al. 2019 (97) | No placebo group (both arms of trial were actively treated). |
| Lin et al. 2016 (98) | Not placebo controlled. |
| Lou et al. 2012 (99) | Not placebo controlled. |
| Lozano et al. 2014 (28) | Not placebo controlled. |
| Maestrelli et al. 2004 (30) | The manufacturer advised that the IMP used in this study, Pangramin Plus SCIT, is no longer commercially available. |
| Maloney et al. 2016 (100) | Study duration was 28 days, less than the threshold of 3 months for inclusion in our review. No clinical outcomes reported. |
| Marcucci et al. 2002 (101) | The manufacturer advised that the IMP used in this study, SlitONE, is no longer commercially available. SlitONE is distinct from SLIToneULTRA, which remains commercially available. |
| Marcucci et al. 2005 (31) | The manufacturer advised that the IMP used in this study, SlitONE, is no longer commercially available. SlitONE is distinct from SLIToneULTRA, which remains commercially available. |
| Marogna et al. 2010 (102) | Not placebo controlled. |
| Matsuoka et al. 2017 (103) | The paper is a secondary analysis of Nolte 2016 and Okubo 2017 which were included individually. |
| Maunsell et al. 1971 (32) | The IMP in this study was presumed to be Bencard Migen; see table A1. The manufacturer advised that Bencard Migen is no longer commercially available. |
| Mauro et al. 2006 (104) | No placebo group (both arms of trial were actively treated). |
| Mungan et al. 1999 (35) | Single-blind study (investigators not blinded); see table A1. |
| Núñez et al. 2000 (105) | The IMP in this study was non-commercial bulk allergen extracts manufactured in Brazil, not a commercially available AIT product, nor is it one of the United States FDA-standardized allergen extracts. |
| Okamiya et al. 2019 (106) | Study duration was 14 days, less than the threshold of 3 months for inclusion in our review. No clinical outcomes reported. |
| Olsen et al. 1997 (107) | Patients in the intervention arm did not all receive the same product. Some received ALK-depot SQ DP 100%, some received ALK-depot DF 100%. Outcomes for each individual product are not presented. ALK-depot SQ is no longer available with 100% DF extract. |
| Pajno et al. 2000 (39) | The manufacturer advised that the IMP used in this study, SlitONE, is no longer commercially available. SlitONE is distinct from SLIToneULTRA, which remains commercially available. |
| Park et al. 2021 (108) | Not a commercially available AIT product. |
| Passàli et al. 2002 (109) | The manufacturer advised that the IMP used in this study, Allerkin Intranasal, is no longer commercially available. |
| Pauli et al. 1984 (40) | The manufacturer advised that the IMP used in this study, Bencard Migen, is no longer commercially available. |
| Peroni et al. 1995 (41) | The manufacturer advised that the IMP used in this study, Alpare Mite, is no longer commercially available. |
| Pichler et al. 1997 (110) | Study did not enroll a homogenous treatment cohort, i.e., some patients had AA but not AR, some had AR but not AA, and some had both. Treatment outcomes are only presented as the entire cohort, not broken down by individual condition. |
| Pifferi et al. 2002 (111) | Not placebo controlled. |
| Queirós et al. 2008 (112) | Not a commercially available AIT product. |
| Queirós et al. 2013 (42) | The AIT formulation in the study was presumed to not be a commercially available product; see table A1. |
| Riechelmann et al. 2010 (43) | This study was of the 100% DP formulation of Purethal Mites. This formulation is no longer available; the commercially available product is 50% DP, 50% DF. |
| Rieker-Schwienbacher et al. 2013 (113) | Not placebo controlled (open label study). |
| Rodriguez et al. 2006 (44) | The manufacturer advised that the IMP in this study, SlitONE, is no longer available. SlitONE was a distinct product from SLIToneULTRA, which is currently available in some markets. |
| Rojas et al. 2015 (114) | Study does not contain a placebo arm; all three arms of the study were actively treated. |
| Rondón et al. 2016 (45) | The manufacturer advised that the IMP used in this study, Pangramin Plus SCIT, is no longer commercially available. |
| Scalone et al. 2013 (115) | No placebo group (both arms of trial were actively treated). |
| Schubert et al. 2009 (116) | No placebo group (both arms of trial were actively treated). |
| Shao et al. 2014 (117) | Not placebo controlled. |
| Smith & Pizarro 1972 (46) | The manufacturer advised that the IMP used in this study, unbranded allergen extracts manufactured by Beecham Research Laboratories, is no longer commercially available. |
| Sobocińska et al. 2012 (118) | Not placebo controlled. |
| Swamy et al. 2012 (119) | Mixed therapeutics used; trial used mixture of Timothy grass and dust mite SLIT. |
| Tabar et al. 2005 (120) | Not placebo controlled. |
| Tabar et al. 2011 (121) | Not placebo controlled. |
| Tabar et al. 2015 (122) | No placebo group (all arms of trial were actively treated). |
| Tahamiler et al. 2008 (123) | No placebo arm (two intervention arms only; one arm received SCIT, the other received SLIT). |
| Tari et al. 1990 (47) | The manufacturer advised that the IMP used in this study, Pangramin Plus SLIT, is no longer commercially available. |
| Tsai et al. 2005 (124) | Not placebo controlled. |
| Tsai et al. 2010 (125) | Not placebo controlled. |
| Valero et al. 2022 (126) | Not placebo controlled. |
| Vesna et al. 2016 (127) | Not placebo controlled (open label study). |
| Vidal et al. 2011 (50) | The manufacturer advised that the IMP used in this study, Pangramin Plus SCIT, is no longer commercially available. |
| Wang et al. 2013 (51) | The manufacturer advised that the IMP used in this study was never commercialized and development has ceased. |
| Wang et al. 2017 (128) | Not placebo controlled. |
| Wang et al. 2018 (129) | Not placebo-controlled (open label trial). |
| Warner et al. 1978 (52) | The manufacturer advised that the IMP used in this study, Bencard Migen, is no longer commercially available. |
| Yin et al. 2016 (130) | Not placebo controlled. |
| Yonekura et al. 2010 (131) | The IMP in the study was a dust extract, not dust mite extract. |
| Zhang et al. 2009 (132) | No placebo group (both arms of trial were actively treated). |
| Zielen et al. 2010 (54) | This paper describes pediatric results. In children, this study was conducted as an open-label study with no placebo group. The adult results, which were placebo-controlled were included (133). |

*IMP, investigational medical product; SCIT, subcutaneous immunotherapy; SLIT, sublingual immunotherapy; AIT, allergen immunotherapy; DP, Dermatophagoides pteronyssinus; DF, Dermatophagoides farinae; AA, allergic asthma; AR, allergic rhinitis; FDA, Food and Drug Administration.*

**Table A3. Studies of currently available allergen immunotherapy products which did not include an arm corresponding to the current manufacturer-recommended maintenance dose the product**

| **Product** | **Dose in Product Information sheet** | **Study** | **Dose used in study** |
| --- | --- | --- | --- |
| Allergopharma allergoid SCIT (Acaroid) | 6,000 TU (0.6 mL of 10,000 TU/mL formulation) SC every 4-8 weeks. | Allergopharma 2015-000188-15 (134) | 18,000 TU (equivalent to 5400 PNU) SC. Dose frequency not stated but presumed to be every 4-8 weeks, as per Acaroid PI. |
| SG 300 IR SLIT tablet (Actair) | 1 x 300 IR tablet SL daily. | Stallergenes-Greer 2013-000487-28 (135) | Three treatment arms: 1 x 100 IR tablet SL daily; 1 x 500 IR tablet SL daily; 1 x 1000 IR tablet SL daily. |
|  |  | Tonnel et al. 2004 (136) | 1 x 100 IR tablet SL daily. |
| SG SLIT liquid (Staloral) | 300 IR (5 x 200 uL sprays of 300 IR/mL solution) every day (seven days per week) | Aydogan et al. 2013(3) | 240 IR (8 x 100 uL sprays of 300 IR/mL solution) SL three days per week. |
|  |  | Bahceciler et al. 2001 (137) | 100 IR (20 x 50 uL drops of 100 IR/mL solution) SL two days per week. |
|  |  | Bousquet et al. 1999 (6) | 300 IR (20 x 50 uL drops of 300 IR/mL solution) SL three days per week. |
|  |  | Bozek et al. 2013 (7) | 540 IR (18 x 50 uL drops of 300 IR/mL solution) SL five days per week. |
|  |  | Guez et al. 2000 (22) | 300 IR (20 x 50 uL drops of 300 IR/mL solution) SL three days per week. |
|  |  | Lue et al. 2006 (29) | 300 IR (20 x 50 uL drops of 300 IR/mL solution) SL, with dosing frequency not stated. Cumulative dose in trial was 41824 IR per patient, lower than the expected 44100 IR if taken daily. |
|  |  | Mortemousqueet al. 2003 (34) | 300 IR (20 x 50 uL drops of 300 IR/mL solution) SL three days per week. |
|  |  | O'Hehir et al. 2009 (38) | 240 IR (8 x 100 uL sprays of 300 IR/mL solution) SL daily (seven days per week). |
|  |  | Potter et al. 2015 (138) | 300 IR (10 x 100 uL sprays of 300 IR/mL solution) SL three days per week. |
|  |  | Tseng et al. 2008 (49) | 300 IR (20 x 50 uL drops of 300 IR/mL solution) SL, with dosing frequency not stated. Cumulative dose in trial was 37312 IR per patient, lower than the expected 44100 IR if taken daily. |
| Lofarma SLIT tablet (Lais) | 1 x 1000 AU tablet SL twice weekly. | Huser et al. 2017 (139) | Four treatment arms: 1 x 300 AU tablet SL daily; 1 x 1000 AU tablet SL daily; 1 x 2000 AU tablet SL daily; 1 x 3000 AU tablet SL daily. |
|  |  | Passalacqua et al. 1998 (140) | 1 x 2000 AU tablet SL twice weekly. |
| HAL Allergy allergoid SCIT (Purethal Mites) | 10,000 AU (0.5 mL of 20,000 AU/mL formulation) SC every 4+/-2 weeks | HAL Allergy 2016-000051-27 (141) | 25,000 AU (0.5 mL of 50,000 AU/mL solution) SC every four weeks. |

*SCIT, subcutaneous immunotherapy; TU, therapeutic unit; SC, subcutaneous; PNU, protein nitrogen unit; PI, product information; SLIT, sublingual immunotherapy; SL, sublingual; IR index of reactivity; AU, allergenic unit.*

**References**

1. Ameal A, Vega-Chicote JM, Fernández S, Miranda A, Carmona MJ, Rondón MC, et al. Double-blind and placebo-controlled study to assess efficacy and safety of a modified allergen extract of Dermatophagoides pteronyssinus in allergic asthma. Allergy. 2005;60(9):1178-83.

2. Andri L, Senna G, Betteli C, Givanni S, Andri G, Falagiani P. Local nasal immunotherapy for Dermatophagoides-induced rhinitis: efficacy of a powder extract. J Allergy Clin Immunol. 1993;91(5):987-96.

3. Aydogan M, Eifan AO, Keles S, Akkoc T, Nursoy MA, Bahceciler NN, et al. Sublingual immunotherapy in children with allergic rhinoconjunctivitis mono-sensitized to house-dust-mites: a double-blind-placebo-controlled randomised trial. Respir Med. 2013;107(9):1322-9.

4. A trial of house dust mite extract in bronchial asthma. Mite Allergy Subcommittee of the Research Committee of the British Thoracic Association. Br J Dis Chest. 1979;73(3):260-70.

5. Blainey AD, Phillips MJ, Ollier S, Davies RJ. Hyposensitization with a tyrosine adsorbed extract of Dermatophagoides pteronyssinus in adults with perennial rhinitis. A controlled clinical trial. Allergy. 1984;39(7):521-8.

6. Bousquet J, Scheinmann P, Guinnepain MT, Perrin-Fayolle M, Sauvaget J, Tonnel AB, et al. Sublingual-swallow immunotherapy (SLIT) in patients with asthma due to house-dust mites: a double-blind, placebo-controlled study. Allergy. 1999;54(3):249-60.

7. Bozek A, Ignasiak B, Filipowska B, Jarzab J. House dust mite sublingual immunotherapy: a double-blind, placebo-controlled study in elderly patients with allergic rhinitis. Clin Exp Allergy. 2013;43(2):242-8.

8. Bożek A, Kołodziejczyk K, Kozłowska R, Canonica GW. Evidence of the efficacy and safety of house dust mite subcutaneous immunotherapy in elderly allergic rhinitis patients: a randomized, double-blind placebo-controlled trial. Clin Transl Allergy. 2017;7:43.

9. Bozek A, Galuszka B, Gawlik R, Misiolek M, Scierski W, Grzanka A, et al. Allergen immunotherapy against house dust mites in patients with local allergic rhinitis and asthma. J Asthma. 2022;59(9):1850-8.

10. Buchanan DJ, Hillis A, Williams PN. A double blind controlled trial of Bencard house dust mite (Migen) hyposensitisation in Zambian asthmatics. Med J Zambia. 1980;15(1):14-6.

11. Bush RK, Swenson C, Fahlberg B, Evans MD, Esch R, Morris M, et al. House dust mite sublingual immunotherapy: results of a US trial. J Allergy Clin Immunol. 2011;127(4):974-81.e1-7.

12. Chen Y, Zhou L, Yang Y. Effect of sublingual immunotherapy on platelet activity in children with allergic rhinitis. Braz J Otorhinolaryngol. 2017;83(2):190-4.

13. de Bot CM, Moed H, Berger MY, Röder E, Hop WC, de Groot H, et al. Sublingual immunotherapy not effective in house dust mite-allergic children in primary care. Pediatr Allergy Immunol. 2012;23(2):150-8.

14. Dokic D, Schnitker J, Narkus A, Cromwell O, Frank E. Clinical effects of specific immunotherapy: a two-year double-blind, placebo-controlled study with a one year follow-up. Prilozi. 2005;26(2):113-29.

15. D'Souza MF, Pepys J, Wells ID, Tai E, Palmer F, Overell BG, et al. Hyposensitization with Dermatophagoides pteronyssinus in house dust allergy: a controlled study of clinical and immunological effects. Clin Allergy. 1973;3(2):177-93.

16. Franco C, Barbadori S, Freshwater LL, Kordash TR. A double-blind, placebo controlled study of Alpare mite D. pteronyssinus immunotherapy in asthmatic patients. Allergol Immunopathol (Madr). 1995;23(2):58-66.

17. Gabriel MS, Ng HK, Allan WG, Hill LE, Nunn AJ. Study of prolonged hyposensitization with D. pteronyssinus extract in allergic rhinitis. Clin Allergy. 1977;7(4):325-39.

18. Gaddie J, Skinner C, Palmer KN. Hyposensitisation with house dust mite vaccine in bronchial asthma. Br Med J. 1976;2(6044):1132-3.

19. García-Robaina JC, Sánchez I, de la Torre F, Fernández-Caldas E, Casanovas M. Successful management of mite-allergic asthma with modified extracts of Dermatophagoides pteronyssinus and Dermatophagoides farinae in a double-blind, placebo-controlled study. J Allergy Clin Immunol. 2006;118(5):1026-32.

20. Giovane AL, Bardare M, Passalacqua G, Ruffoni S, Scordamaglia A, Ghezzi E, et al. A three-year double-blind placebo-controlled study with specific oral immunotherapy to Dermatophagoides: evidence of safety and efficacy in paediatric patients. Clin Exp Allergy. 1994;24(1):53-9.

21. Grembiale RD, Camporota L, Naty S, Tranfa CM, Djukanovic R, Marsico SA. Effects of specific immunotherapy in allergic rhinitic individuals with bronchial hyperresponsiveness. Am J Respir Crit Care Med. 2000;162(6):2048-52.

22. Guez S, Vatrinet C, Fadel R, Andre C. House-dust-mite sublingual-swallow immunotherapy (SLIT) in perennial rhinitis: a double-blind, placebo-controlled study. Allergy. 2000;55(4):369-75.

23. Guo Y, Li Y, Wang D, Liu Q, Liu Z, Hu L. A randomized, double-blind, placebo controlled trial of sublingual immunotherapy with house-dust mite extract for allergic rhinitis. Am J Rhinol Allergy. 2017;31(4):42-7.

24. EudraCT 2016-000051-27. A randomized, double-blind, placebo-controlled (DBPC) parallel-group multi-centre study to assess the efficacy and safety of PM subcutaneous immunotherapy (SCIT) in patients with allergic rhinitis/rhinoconjunctivitis (ARC) caused by house dust mite (HDM) allergy [Internet]. 2016 [cited 30 Jun 2023]. Available from: <https://www.clinicaltrialsregister.eu/ctr-search/trial/2016-000051-27/results>.

25. Hoseini RF, Jabbari F, Rezaee A, Rafatpanah H, Yousefzadeh H, Ariaee N, et al. House dust mite sublingual-swallow immunotherapy in perennial rhinitis: a double-blind, placebo-controlled Iranian study. J Biol Regul Homeost Agents. 2018;32(1):83-8.

26. Ippoliti F, De Santis W, Volterrani A, Lenti L, Canitano N, Lucarelli S, et al. Immunomodulation during sublingual therapy in allergic children. Pediatr Allergy Immunol. 2003;14(3):216-21.

27. Jutel M, Rudert M, Kreimendahl F, Kuna P. Efficacy and tolerability of a house dust mite allergoid in allergic bronchial asthma: a randomized dose-ranging trial. Immunotherapy. 2018;10(13):1149-61.

28. Lozano J, Cruz MJ, Piquer M, Giner MT, Plaza AM. Assessing the efficacy of immunotherapy with a glutaraldehyde-modified house dust mite extract in children by monitoring changes in clinical parameters and inflammatory markers in exhaled breath. Int Arch Allergy Immunol. 2014;165(2):140-7.

29. Lue KH, Lin YH, Sun HL, Lu KH, Hsieh JC, Chou MC. Clinical and immunologic effects of sublingual immunotherapy in asthmatic children sensitized to mites: a double-blind, randomized, placebo-controlled study. Pediatr Allergy Immunol. 2006;17(6):408-15.

30. Maestrelli P, Zanolla L, Pozzan M, Fabbri LM. Effect of specific immunotherapy added to pharmacologic treatment and allergen avoidance in asthmatic patients allergic to house dust mite. J Allergy Clin Immunol. 2004;113(4):643-9.

31. Marcucci F, Sensi L, Di Cara G, Salvatori S, Bernini M, Pecora S, et al. Three-year follow-up of clinical and inflammation parameters in children monosensitized to mites undergoing sub-lingual immunotherapy. Pediatr Allergy Immunol. 2005;16(6):519-26.

32. Maunsell K, Wraith DG, Hughes AM. Hyposensitisation in mite asthma. Lancet. 1971;1(7706):967-8.

33. Moreno V, Alvarino M, Rodriguez F, Roger A, Pena-Arellano MI, Lleonart R, et al. Randomized dose-response study of subcutaneous immunotherapy with a Dermatophagoides pteronyssinus extract in patients with respiratory allergy. Immunotherapy. 2016;8(3):265-77.

34. Mortemousque B, Bertel F, De Casamayor J, Verin P, Colin J. House-dust mite sublingual-swallow immunotherapy in perennial conjunctivitis: a double-blind, placebo-controlled study. Clin Exp Allergy. 2003;33(4):464-9.

35. Mungan D, Misirligil Z, Gürbüz L. Comparison of the efficacy of subcutaneous and sublingual immunotherapy in mite-sensitive patients with rhinitis and asthma--a placebo controlled study. Ann Allergy Asthma Immunol. 1999;82(5):485-90.

36. Nieto A, Mazón Á, Nieto M, Ibáñez E, Jang DT, Calaforra S, et al. First-in-human phase 2 trial with mite allergoids coupled to mannan in subcutaneous and sublingual immunotherapy. Allergy. 2022;77(10):3096-107.

37. Niu CK, Chen WY, Huang JL, Lue KH, Wang JY. Efficacy of sublingual immunotherapy with high-dose mite extracts in asthma: a multi-center, double-blind, randomized, and placebo-controlled study in Taiwan. Respir Med. 2006;100(8):1374-83.

38. O'Hehir RE, Gardner LM, de Leon MP, Hales BJ, Biondo M, Douglass JA, et al. House dust mite sublingual immunotherapy: the role for transforming growth factor-beta and functional regulatory T cells. Am J Respir Crit Care Med. 2009;180(10):936-47.

39. Pajno GB, Morabito L, Barberio G, Parmiani S. Clinical and immunologic effects of long-term sublingual immunotherapy in asthmatic children sensitized to mites: a double-blind, placebo-controlled study. Allergy. 2000;55(9):842-9.

40. Pauli G, Bessot JC, Bigot H, Delaume G, Hordle DA, Hirth C, et al. Clinical and immunologic evaluation of tyrosine-adsorbed Dermatophagoides pteronyssinus extract: a double-blind placebo-controlled trial. J Allergy Clin Immunol. 1984;74(4 Pt 1):524-35.

41. Peroni DG, Piacentini GL, Martinati LC, Warner JO, Boner AL. Double-blind trial of house-dust mite immunotherapy in asthmatic children resident at high altitude. Allergy. 1995;50(11):925-30.

42. Queirós MG, Silva DA, Siman IL, Ynoue LH, Araújo NS, Pereira FL, et al. Modulation of mucosal/systemic antibody response after sublingual immunotherapy in mite-allergic children. Pediatr Allergy Immunol. 2013;24(8):752-61.

43. Riechelmann H, Schmutzhard J, van der Werf JF, Distler A, Kleinjans HA. Efficacy and safety of a glutaraldehyde-modified house dust mite extract in allergic rhinitis. Am J Rhinol Allergy. 2010;24(5):e104-9.

44. Rodriguez F, Boquete M, Ibáñez MD, de la Torre-Martínez F, Tabar AI. Once daily sublingual immunotherapy without updosing--A new treatment schedule. Int Arch Allergy Immunol. 2006;140(4):321-6.

45. Rondón C, Campo P, Salas M, Aranda A, Molina A, González M, et al. Efficacy and safety of D. pteronyssinus immunotherapy in local allergic rhinitis: a double-blind placebo-controlled clinical trial. Allergy. 2016;71(7):1057-61.

46. Smith JM, Pizarro YA. Hyposensitization with extracts of Dermatophagoides pteronyssinus and house dust. Clin Allergy. 1972;2(3):281-3.

47. Tari MG, Mancino M, Monti G. Efficacy of sublingual immunotherapy in patients with rhinitis and asthma due to house dust mite. A double-blind study. Allergol Immunopathol (Madr). 1990;18(5):277-84.

48. Tian M, Wang Y, Lu Y, Jiang YH, Zhao DY. Effects of sublingual immunotherapy for Dermatophagoides farinae on Th17 cells and CD4(+) CD25(+) regulatory T cells in peripheral blood of children with allergic asthma. Int Forum Allergy Rhinol. 2014;4(5):371-5.

49. Tseng SH, Fu LS, Nong BR, Weng JD, Shyur SD. Changes in serum specific IgG4 and IgG4/ IgE ratio in mite-sensitized Taiwanese children with allergic rhinitis receiving short-term sublingual-swallow immunotherapy: a multicenter, randomized, placebo-controlled trial. Asian Pac J Allergy Immunol. 2008;26(2-3):105-12.

50. Vidal C, Tabar AI, Figueroa J, Navarro JA, Sánchez C, Orovitg A, et al. Assessment of short-term changes induced by a Dermatophagoides pteronyssinus extract on asthmatic patients. Randomised, double-blind, placebo-controlled trial. Curr Drug Deliv. 2011;8(2):152-8.

51. Wang DH, Chen L, Cheng L, Li KN, Yuan H, Lu JH, et al. Fast onset of action of sublingual immunotherapy in house dust mite-induced allergic rhinitis: a multicenter, randomized, double-blind, placebo-controlled trial. Laryngoscope. 2013;123(6):1334-40.

52. Warner JO, Price JF, Soothill JF, Hey EN. Controlled trial of hyposensitisation to Dermatophagoides pteronyssinus in children with asthma. Lancet. 1978;2(8096):912-5.

53. Yukselen A, Kendirli SG, Yilmaz M, Altintas DU, Karakoc GB. Effect of one-year subcutaneous and sublingual immunotherapy on clinical and laboratory parameters in children with rhinitis and asthma: a randomized, placebo-controlled, double-blind, double-dummy study. Int Arch Allergy Immunol. 2012;157(3):288-98.

54. Zielen S, Kardos P, Madonini E. Steroid-sparing effects with allergen-specific immunotherapy in children with asthma: a randomized controlled trial. J Allergy Clin Immunol. 2010;126(5):942-9.

55. Jutel M, Zieglmayer P, Willers C, editors. Results from a phase III trial investigating subcutaneous

immunotherapy with a house dust mite allergoid in patients with

HDM induced asthma and allergic rhinitis/rhinoconjunctivitis (abstract 1890). EAACI Digital Congress 2020; 2020: Allergy.

56. EudraCT 2005-004731-21. A randomised, double-blind, placebo-controlled trial assessing the efficacy of SLITone in house dust mite allergic patients [Internet]. EU Clinical Trials Register. 2005 [cited 30 Jun 2023]. Available from: <https://www.clinicaltrialsregister.eu/ctr-search/trial/2005-004731-21/ES>.

57. NCT01603056. Efficacy and Safety Trial of Pangramin SLIT HDM-mix in Subjects With House Dust Mite Induced Rhinitis. [Internet]. 2012 [cited 30 Jun 2023]. Available from: <https://classic.clinicaltrials.gov/ct2/show/study/NCT01603056>.

58. Alvarez MJ, Echechipía S, García B, Tabar AI, Martín S, Rico P, et al. Liposome-entrapped D. pteronyssinus vaccination in mild asthma patients: effect of 1-year double-blind, placebo-controlled trial on inflammation, bronchial hyperresponsiveness and immediate and late bronchial responses to the allergen. Clin Exp Allergy. 2002;32(11):1574-82.

59. Arikan C, Bahceciler NN, Deniz G, Akdis M, Akkoc T, Akdis CA, et al. Bacillus Calmette-Guérin-induced interleukin-12 did not additionally improve clinical and immunologic parameters in asthmatic children treated with sublingual immunotherapy. Clin Exp Allergy. 2004;34(3):398-405.

60. Baris S, Kiykim A, Ozen A, Tulunay A, Karakoc-Aydiner E, Barlan IB. Vitamin D as an adjunct to subcutaneous allergen immunotherapy in asthmatic children sensitized to house dust mite. Allergy. 2014;69(2):246-53.

61. Basomba A, Tabar AI, de Rojas DH, García BE, Alamar R, Olaguíbel JM, et al. Allergen vaccination with a liposome-encapsulated extract of Dermatophagoides pteronyssinus: a randomized, double-blind, placebo-controlled trial in asthmatic patients. J Allergy Clin Immunol. 2002;109(6):943-8.

62. Bousquet J, Calvayrac P, Guérin B, Hejjaoui A, Dhivert H, Hewitt B, et al. Immunotherapy with a standardized Dermatophagoides pteronyssinus extract. I. In vivo and in vitro parameters after a short course of treatment. J Allergy Clin Immunol. 1985;76(5):734-44.

63. Branco Ferreira M, Spínola Santos A, Pereira Santos MC, Palma Carlos ML, Pereira Barbosa MA, Palma Carlos AG. Efficacy and safety of specific immunotherapy with a modified mite extract. Allergol Immunopathol (Madr). 2005;33(2):80-5.

64. Casanovas M, Fernández-Caldas E, Alamar R, Basomba A. Comparative study of tolerance between unmodified and high doses of chemically modified allergen vaccines of Dermatophagoides pteronyssinus. Int Arch Allergy Immunol. 2005;137(3):211-8.

65. Chen ZG, Li M, Chen YF, Ji JZ, Li YT, Chen W, et al. Effects of dermatophagoides pteronyssinus allergen-specific immunotherapy on the serum interleukin-13 and pulmonary functions in asthmatic children. Chin Med J (Engl). 2009;122(10):1157-61.

66. Chen WB, Shen XF, Li Q, Zhou WC, Cheng L. Efficacy of a 3-year course of sublingual immunotherapy for mite-induced allergic rhinitis with a 3-year follow-up. Immunotherapy. 2020;12(12):891-901.

67. Chen H, Chen Y, Lin B, Huang S, Liu Q, Zheng H, et al. Efficacy and adherence of sublingual immunotherapy in patients aged 60 to 75 years old with house dust mite-induced allergic rhinitis. Am J Otolaryngol. 2020;41(4):102538.

68. Chen H, Tian J, Xu H, Yang Y. The effects of sublingual specific immunization with dermatophagoides farinae drops on asthma and serum CD4+ and CD8+ cell count and IgE levels in children. Int J Clin Exp Med. 2020;13(6):4211-8.

69. EudraCT 2014-001662-94. A Double-Blind, Randomised, Placebo-Controlled, Multi-Centre Field Study to Assess the Efficacy and Safety of HDM-SPIRE in Subjects with a History of House Dust Mite-Induced Rhinoconjunctivitis [Internet]. 2014 [cited 30 Jun 2023]. Available from: <https://www.clinicaltrialsregister.eu/ctr-search/search?query=2014-001662-94>.

70. NIOX. Circassia announces top line results from house dust mite allergy field study: NIOX; 2022 [Available from: <https://www.circassia.com/media/press-releases/circassia-announces-top-line-results-from-house-dust-mite-allergy-field-study/>.

71. Corzo JL, Carrillo T, Pedemonte C, Plaza Martin AM, Martin Hurtado S, Dige E, et al. Tolerability during double-blind randomized phase I trials with the house dust mite allergy immunotherapy tablet in adults and children. J Investig Allergol Clin Immunol. 2014;24(3):154-61.

72. Cosmi L, Santarlasci V, Angeli R, Liotta F, Maggi L, Frosali F, et al. Sublingual immunotherapy with Dermatophagoides monomeric allergoid down-regulates allergen-specific immunoglobulin E and increases both interferon-gamma- and interleukin-10-production. Clin Exp Allergy. 2006;36(3):261-72.

73. Didier A, Campo P, Moreno F, Durand-Perdriel F, Marin A, Chartier A. Dose-Dependent Immunological Responses after a 6-Month Course of Sublingual House Dust Mite Immunotherapy in Patients with Allergic Rhinitis. Int Arch Allergy Immunol. 2015;168(3):182-92.

74. Dreborg S, Lee TH, Kay AB, Durham SR. Immunotherapy is allergen-specific: a double-blind trial of mite or timothy extract in mite and grass dual-allergic patients. Int Arch Allergy Immunol. 2012;158(1):63-70.

75. Eifan AO, Akkoc T, Yildiz A, Keles S, Ozdemir C, Bahceciler NN, et al. Clinical efficacy and immunological mechanisms of sublingual and subcutaneous immunotherapy in asthmatic/rhinitis children sensitized to house dust mite: an open randomized controlled trial. Clin Exp Allergy. 2010;40(6):922-32.

76. Fan Q, Liu X, Gao J, Huang S, Ni L. Comparative analysis of cluster versus conventional immunotherapy in patients with allergic rhinitis. Exp Ther Med. 2017;13(2):717-22.

77. Fanales-Belasio E, Ciofalo A, Zambetti G, Ansotegui IJ, Scala E, Paganelli R, et al. Intranasal immunotherapy with Dermatophagoides extract: in vivo and in vitro results of a double-blind placebo-controlled trial. Rhinology. 1995;33(3):126-31.

78. Feng M, Zeng X, Su Q, Shi X, Xian M, Qin R, et al. Allergen Immunotherapy-Induced Immunoglobulin G4 Reduces Basophil Activation in House Dust Mite-Allergic Asthma Patients. Front Cell Dev Biol. 2020;8:30.

79. Feng M, Luo T, Xian M, Shi X, Qin R, Zeng X, et al. Suppression function against environmental dust exposure after Dermatophagoides pteronyssinus immunotherapy is associated with production of specific and cross-reactive immunoglobulin G4. Clin Exp Allergy. 2022;52(7):878-87.

80. Ferreira MB, Santos AS, Santos MC, Carlos ML, Barbosa MA, Carlos AG. Nasal ECP patterns and specific immunotherapy in mite-allergic rhinitis patients. Eur Ann Allergy Clin Immunol. 2005;37(3):96-102.

81. Ferrer A, García-Sellés J. Significant improvement in symptoms, skin test, and specific bronchial reactivity after 6 months of treatment with a depigmented, polymerized extract of Dermatophagoides pteronyssinus and D. farinae. J Investig Allergol Clin Immunol. 2003;13(4):244-51.

82. Gardner LM, Thien FC, Douglass JA, Rolland JM, O'Hehir RE. Induction of T 'regulatory' cells by standardized house dust mite immunotherapy: an increase in CD4+ CD25+ interleukin-10+ T cells expressing peripheral tissue trafficking markers. Clin Exp Allergy. 2004;34(8):1209-19.

83. Di Gioacchino M, Cavallucci E, Ballone E, Cervone M, Di Rocco P, Piunti E, et al. Dose-dependent clinical and immunological efficacy of sublingual immunotherapy with mite monomeric allergoid. Int J Immunopathol Pharmacol. 2012;25(3):671-9.

84. Hoshino M, Akitsu K, Kubota K. Effect of Sublingual Immunotherapy on Airway Inflammation and Airway Wall Thickness in Allergic Asthma. J Allergy Clin Immunol Pract. 2019;7(8):2804-11.

85. Hoshino M, Akitsu K, Kubota K, Ohtawa J. Association between biomarkers and house dust mite sublingual immunotherapy in allergic asthma. Clin Exp Allergy. 2020;50(9):1035-43.

86. Hoshino M, Akitsu K, Kubota K, Ohtawa J. Serum Periostin as a Biomarker for Predicting Clinical Response to House Dust Mite Sublingual Immunotherapy in Allergic Rhinitis. J Allergy Clin Immunol Pract. 2021;9(5):1864-70.

87. Hui Y, Li L, Qian J, Guo Y, Zhang X, Zhang X. Efficacy analysis of three-year subcutaneous SQ-standardized specific immunotherapy in house dust mite-allergic children with asthma. Exp Ther Med. 2014;7(3):630-4.

88. Ibero M, Castillo MJ. Significant improvement of specific bronchial hyperreactivity in asthmatic children after 4 months of treatment with a modified extract of dermatophagoides pteronyssinus. J Investig Allergol Clin Immunol. 2006;16(3):194-202.

89. Keles S, Karakoc-Aydiner E, Ozen A, Izgi AG, Tevetoglu A, Akkoc T, et al. A novel approach in allergen-specific immunotherapy: combination of sublingual and subcutaneous routes. J Allergy Clin Immunol. 2011;128(4):808-15.e7.

90. Kim JH, Lee JH, Ye YM, Lee JH, Park JW, Hur GY, et al. Efficacy and Safety of Sublingual Immunotherapy in Elderly Rhinitis Patients Sensitized to House Dust Mites. Allergy Asthma Immunol Res. 2018;10(6):675-85.

91. Kim CK, Callaway Z, Park JS, Kwon E. Efficacy of subcutaneous immunotherapy for patients with asthma and allergic rhinitis in Korea: effect on eosinophilic inflammation. Asia Pac Allergy. 2021;11(4):e43.

92. Królewicz E, Wolańczyk-Mędrala A, Gomułka K, Mędrala W, Barg W. A pilot study on house dust mites’ allergens in south-western Poland - impact of allergen-specific immunotherapy. nternational Review of Allergology and Clinical Immunology in Family Medicine. 2016;22:211-4.

93. La Grutta S, Arena A, D'Anneo WR, Gammeri E, Leonardi S, Trimarchi A, et al. Evaluation of the antiinflammatory and clinical effects of sublingual immunotherapy with carbamylated allergoid in allergic asthma with or without rhinitis. A 12-month perspective randomized, controlled, trial. Eur Ann Allergy Clin Immunol. 2007;39(2):40-4.

94. Lauriello M, Muzi P, Di Rienzo L, Di Stanislao C, Tirelli GC, Bologna M. A two-year course of specific immunotherapy or of continuous antihistamine treatment reverse eosinophilic inflammation in severe persistent allergic rhinitis. Acta Otorhinolaryngol Ital. 2005;25(5):284-91.

95. Lewith GT, Watkins AD, Hyland ME, Shaw S, Broomfield JA, Dolan G, et al. Use of ultramolecular potencies of allergen to treat asthmatic people allergic to house dust mite: double blind randomised controlled clinical trial. Bmj. 2002;324(7336):520.

96. Li H, Yang P, Chen X, Sun W, Qu D, Zhao X, et al. A comparative study of sublingual and subcutaneous immunotherapy in mite-sensitive asthmatic children: a single center experience of 90 Chinese patients. Int J Clin Exp Med. 2016;9(3):6743-50.

97. Li J, Wu Y, Yang Y, Huang N, Li W, Zhang S, et al. The efficacy and safety of two commercial house dust mite extracts for allergic rhinitis: a head-to-head study. Int Forum Allergy Rhinol. 2019;9(8):876-82.

98. Lin Z, Liu Q, Li T, Chen D, Chen D, Xu R. The effects of house dust mite sublingual immunotherapy in patients with allergic rhinitis according to duration. Int Forum Allergy Rhinol. 2016;6(1):82-7.

99. Lou W, Wang C, Wang Y, Han D, Zhang L. Responses of CD4(+) CD25(+) Foxp3(+) and IL-10-secreting type I T regulatory cells to cluster-specific immunotherapy for allergic rhinitis in children. Pediatr Allergy Immunol. 2012;23(2):140-9.

100. Maloney J, Prenner BM, Bernstein DI, Lu S, Gawchik S, Berman G, et al. Safety of house dust mite sublingual immunotherapy standardized quality tablet in children allergic to house dust mites. Ann Allergy Asthma Immunol. 2016;116(1):59-65.

101. Marcucci F, Sensi LG, Caffarelli C, Cavagni G, Bernardini R, Tiri A, et al. Low-dose local nasal immunotherapy in children with perennial allergic rhinitis due to Dermatophagoides. Allergy. 2002;57(1):23-8.

102. Marogna M, Colombo F, Cerra C, Bruno M, Massolo A, Canonica GW, et al. The clinical efficacy of a sublingual monomeric allergoid at different maintenance doses: a randomized controlled trial. Int J Immunopathol Pharmacol. 2010;23(3):937-45.

103. Matsuoka T, Bernstein DI, Masuyama K, Nolte H, Okamiya K, Seitzberg D, et al. Pooled efficacy and safety data for house dust mite sublingual immunotherapy tablets in adolescents. Pediatr Allergy Immunol. 2017;28(7):661-7.

104. Mauro M, Russello M, Alesina R, Sillano V, Alessandrini A, Dama A, et al. Safety and pharmacoeconomics of a cluster administration of mite immunotherapy compared to the traditional one. Eur Ann Allergy Clin Immunol. 2006;38(1):31-4.

105. Núñez JA, Cuesta U. Local conjunctival immunotherapy: the effect of dermatophagoides pteronyssinus local conjunctival immunotherapy on conjunctival provocation test in patients with allergic conjunctivitis. Allergol Immunopathol (Madr). 2000;28(6):301-6.

106. Okamiya K, Sekino H, Azuma R, Kudo M, Sakaguchi M, Nemoto F, et al. Safety profile of the SQ house dust mite sublingual immunotherapy-tablet in Japanese adult patients with house dust mite-induced allergic asthma: a randomized, double-blind, placebo-controlled phase I study. J Asthma. 2019;56(12):1347-55.

107. Olsen OT, Larsen KR, Jacobsan L, Svendsen UG. A 1-year, placebo-controlled, double-blind house-dust-mite immunotherapy study in asthmatic adults. Allergy. 1997;52(8):853-9.

108. Park HJ, Kim SH, Shin YS, Park CH, Cho ES, Choi SJ, et al. Intralymphatic immunotherapy with tyrosine-adsorbed allergens: a double-blind, placebo-controlled trial. Respir Res. 2021;22(1):170.

109. Passali D, Bellussi L, Passali GC, Passali FM. Nasal immunotherapy is effective in the treatment of rhinitis due to mite allergy. A double-blind, placebo-controlled study with rhinological evaluation. Int J Immunopathol Pharmacol. 2002;15(2):141-7.

110. Pichler CE, Marquardsen A, Sparholt S, Løwenstein H, Bircher A, Bischof M, et al. Specific immunotherapy with Dermatophagoides pteronyssinus and D. farinae results in decreased bronchial hyperreactivity. Allergy. 1997;52(3):274-83.

111. Pifferi M, Baldini G, Marrazzini G, Baldini M, Ragazzo V, Pietrobelli A, et al. Benefits of immunotherapy with a standardized Dermatophagoides pteronyssinus extract in asthmatic children: a three-year prospective study. Allergy. 2002;57(9):785-90.

112. Guimarães Junqueir de Queirós M, Oliveira Silva DA, Alves R, Fukuhara Chiba H, Soares de Amaral VB, de Almeida KC, et al. Mite-specific immunotherapy using allergen and/or bacterial extracts in atopic patients in Brazil. J Investig Allergol Clin Immunol. 2008;18(2):84-92.

113. Rieker-Schwienbacher J, Nell MJ, Diamant Z, van Ree R, Distler A, Boot JD, et al. Open-label parallel dose tolerability study of three subcutaneous immunotherapy regimens in house dust mite allergic patients. Clin Transl Allergy. 2013;3(1):16.

114. Hernández Fernández de Rojas D, Antépara Ercoreca I, Ponte Tellechea A, Ibáñez Echevarría E, Jáuregui Presa I, Gamboa Setién P, et al. Phase I study of subcutaneous allergen immunotherapy with Dermatophagoides pteronyssinus in patients with allergic rhinoconjunctivitis with or without asthma. Immunotherapy. 2015;7(2):89-99.

115. Scalone G, Compalati E, Bruno ME, Mistrello G. Effect of two doses of carbamylated allergoid extract of dust mite on nasal reactivity. Eur Ann Allergy Clin Immunol. 2013;45(6):193-200.

116. Schubert R, Eickmeier O, Garn H, Baer PC, Mueller T, Schulze J, et al. Safety and immunogenicity of a cluster specific immunotherapy in children with bronchial asthma and mite allergy. Int Arch Allergy Immunol. 2009;148(3):251-60.

117. Shao J, Cui YX, Zheng YF, Peng HF, Zheng ZL, Chen JY, et al. Efficacy and safety of sublingual immunotherapy in children aged 3-13 years with allergic rhinitis. Am J Rhinol Allergy. 2014;28(2):131-9.

118. Sobocińska A, Majak P, Jerzyńska J, Ozarek-Hanc A. Early effectiveness of allergen immunotherapy in children with asthma allergic to house dust mite. Alergia Astma Immunologia. 2012;17(1):27-31.

119. Swamy RS, Reshamwala N, Hunter T, Vissamsetti S, Santos CB, Baroody FM, et al. Epigenetic modifications and improved regulatory T-cell function in subjects undergoing dual sublingual immunotherapy. J Allergy Clin Immunol. 2012;130(1):215-24.e7.

120. Tabar AI, Echechipía S, García BE, Olaguibel JM, Lizaso MT, Gómez B, et al. Double-blind comparative study of cluster and conventional immunotherapy schedules with Dermatophagoides pteronyssinus. J Allergy Clin Immunol. 2005;116(1):109-18.

121. Tabar AI, Arroabarren E, Echechipía S, García BE, Martin S, Alvarez-Puebla MJ. Three years of specific immunotherapy may be sufficient in house dust mite respiratory allergy. J Allergy Clin Immunol. 2011;127(1):57-63, .e1-3.

122. Tabar AI, González Delgado P, Sánchez Hernández C, Basagaña Torrento M, Moreno Benítez F, Arina M. Phase II/III clinical trial to assess the tolerability and immunological effect of a new updosing phase of Dermatophagoides mix-based immunotherapy. J Investig Allergol Clin Immunol. 2015;25(1):40-6.

123. Tahamiler R, Saritzali G, Canakcioglu S, Ozcora E, Dirican A. Comparison of the long-term efficacy of subcutaneous and sublingual immunotherapies in perennial rhinitis. ORL J Otorhinolaryngol Relat Spec. 2008;70(3):144-50.

124. Tsai YG, Chien JW, Chen WL, Shieh JJ, Lin CY. Induced apoptosis of TH2 lymphocytes in asthmatic children treated with Dermatophagoides pteronyssinus immunotherapy. Pediatr Allergy Immunol. 2005;16(7):602-8.

125. Tsai TC, Lu JH, Chen SJ, Tang RB. Clinical efficacy of house dust mite-specific immunotherapy in asthmatic children. Pediatr Neonatol. 2010;51(1):14-8.

126. Valero A, Ibáñez-Echevarría E, Vidal C, Raducan I, Castelló Carrascosa JV, Sánchez-López J. Efficacy of subcutaneous house dust mite immunotherapy in patients with moderate to severe allergic rhinitis. Immunotherapy. 2022;14(9):683-94.

127. Vesna TS, Denisa D, Slavenka J, Lidija B, Aleksandra B, Jasna B, et al. Efficacy of Sublingual Immunotherapy with Dermatophagoides Pteronyssinus: A Real-life Study. Iran J Allergy Asthma Immunol. 2016;15(2):112-21.

128. Wang D, Zhang E, Xiao E. Effect of specific immunotherapy induced by Dermatophagoides farinae on treatment of children with combined allergic rhinitis and asthma syndrome. Biomedical Research. 2017;28(20):8909-12.

129. Wang Y, Li C, Xu Y, Xu D, Yang G, Liao F, et al. Sublingual Immunotherapy Decreases Expression of Interleukin-33 in Children with Allergic Rhinitis. Indian J Pediatr. 2018;85(10):872-6.

130. Yin GQ, Jiang WH, Wu PQ, He CH, Chen RS, Deng L. Clinical evaluation of sublingual administration of dust mite drops in the treatment of allergic asthma and allergic rhinitis of children. Eur Rev Med Pharmacol Sci. 2016;20(20):4348-53.

131. Yonekura S, Okamoto Y, Sakurai D, Horiguchi S, Hanazawa T, Nakano A, et al. Sublingual immunotherapy with house dust extract for house dust-mite allergic rhinitis in children. Allergol Int. 2010;59(4):381-8.

132. Zhang L, Wang C, Han D, Wang X, Zhao Y, Liu J. Comparative study of cluster and conventional immunotherapy schedules with dermatophagoides pteronyssinus in the treatment of persistent allergic rhinitis. Int Arch Allergy Immunol. 2009;148(2):161-9.

133. KG. AGC. A multicentre randomised placebo-controlled double-blind clinical trial for evaluation of safety and efficacy of specific immunotherapy with an aluminium hydroxide-adsorbed Allergoid Preparation of house dust mite (Dermatophagoides pteronyssinus) in patients with allergic asthma bronchiale +/- rhinitis / rhinoconjunctivitis (EudraCT 2004-003892-35): EU Clinical Trials Register; 2018 [Available from: <https://www.clinicaltrialsregister.eu/ctr-search/trial/2004-003892-35/results>.

134. KG AGC. A multicenter randomized double-blind placebo-controlled clinical trial for evaluation of efficacy and safety of specific immunotherapy with an aluminium hydroxide-adsorbed allergoid preparation of house dust mite (Dermatophagoides pteronyssinus) in patients with allergic bronchial asthma and with allergic rhinitis or rhinoconjunctivitis (EudraCT 2015-000188-15): EU Clinical Trials Register; 2020 [Available from: <https://www.clinicaltrialsregister.eu/ctr-search/trial/2015-000188-15/results>.

135. SA S. A dose ranging study investigating the efficacy and safety of sublingual immunotherapy tablets of house dust mite allergen extracts in adults with house dust mite-associated allergic asthma (EudraCT 2013-000487-28): EU Clinical Trials Register ; 2013 [Available from: <https://www.clinicaltrialsregister.eu/ctr-search/search?query=2013-000487-28>.

136. Tonnel AB, Scherpereel A, Douay B, Mellin B, Leprince D, Goldstein N, et al. Allergic rhinitis due to house dust mites: evaluation of the efficacy of specific sublingual immunotherapy. Allergy. 2004;59(5):491-7.

137. Bahceciler NN, Isik U, Barlan IB, Basaran MM. Efficacy of sublingual immunotherapy in children with asthma and rhinitis: a double-blind, placebo-controlled study. Pediatr Pulmonol. 2001;32(1):49-55.

138. Potter PC, Baker S, Fenemore B, Nurse B. Clinical and cytokine responses to house dust mite sublingual immunotherapy. Ann Allergy Asthma Immunol. 2015;114(4):327-34.

139. Hüser C, Dieterich P, Singh J, Shah-Hosseini K, Allekotte S, Lehmacher W, et al. A 12-week DBPC dose-finding study with sublingual monomeric allergoid tablets in house dust mite-allergic patients. Allergy. 2017;72(1):77-84.

140. Passalacqua G, Albano M, Fregonese L, Riccio A, Pronzato C, Mela GS, et al. Randomised controlled trial of local allergoid immunotherapy on allergic inflammation in mite-induced rhinoconjunctivitis. Lancet. 1998;351(9103):629-32.

141. B.V. HA. A randomized, double-blind, placebo-controlled (DBPC) parallel-group multi-centre study to assess the efficacy and safety of PM subcutaneous immunotherapy (SCIT) in patients with allergic rhinitis/rhinoconjunctivitis (ARC) caused by house dust mite (HDM) allergy (EudraCT 2016-000051-27): EU Clinical Trials Register; 2021 [Available from: <https://www.clinicaltrialsregister.eu/ctr-search/trial/2016-000051-27/results>.
